# Noble metal-free bifunctional oxygen evolution and oxygen reduction acidic media electro-catalysts

**DOI:** 10.1038/srep28367

**Published:** 2016-07-06

**Authors:** Prasad Prakash Patel, Moni Kanchan Datta, Oleg I. Velikokhatnyi, Ramalinga Kuruba, Krishnan Damodaran, Prashanth Jampani, Bharat Gattu, Pavithra Murugavel Shanthi, Sameer S. Damle, Prashant N. Kumta

**Affiliations:** 1 Department of Chemical and Petroleum Engineering, Swanson School of Engineering, University of Pittsburgh, Pittsburgh, PA 15261, USA; 2 Department of Bioengineering, Swanson School of Engineering, University of Pittsburgh, Pittsburgh, PA 15261, USA; 3 Center for Complex Engineered Multifunctional Materials, University of Pittsburgh, PA 15261, USA; 4 Department of Chemistry, University of Pittsburgh, PA 15260, USA; 5 Mechanical Engineering and Materials Science, Swanson School of Engineering, University of Pittsburgh, Pittsburgh, PA 15261, USA; 6 School of Dental Medicine, University of Pittsburgh, PA 15217, USA

## Abstract

Identification of low cost, highly active, durable completely noble metal-free electro-catalyst for oxygen reduction reaction (ORR) in proton exchange membrane (PEM) fuel cells, oxygen evolution reaction (OER) in PEM based water electrolysis and metal air batteries remains one of the major unfulfilled scientific and technological challenges of PEM based acid mediated electro-catalysts. In contrast, several non-noble metals based electro-catalysts have been identified for alkaline and neutral medium water electrolysis and fuel cells. Herein we report for the very first time, F doped Cu_1.5_Mn_1.5_O_4_, identified by exploiting theoretical first principles calculations for ORR and OER in PEM based systems. The identified novel noble metal-free electro-catalyst showed similar onset potential (1.43 V for OER and 1 V for ORR *vs* RHE) to that of IrO_2_ and Pt/C, respectively. The system also displayed excellent electrochemical activity comparable to IrO_2_ for OER and Pt/C for ORR, respectively, along with remarkable long term stability for 6000 cycles in acidic media validating theory, while also displaying superior methanol tolerance and yielding recommended power densities in full cell configurations.

The design and development of economic noble metal-free electro-catalysts with comparable or superior electrochemical performance and stability than noble metal or noble metal oxide based electro-catalysts is a major research area for the sustainable energy future of the world with massive technological and economic gains^1^. The performance and efficiency of energy generation and storage technologies is highly dependent on the nature of the electro-catalyst. Highly efficient and cost-effective electro-catalysts are very much desirable if fuel cells^2^,^3^,^4^, hydrogen generation from solar and electrolytic water splitting^5^ and metal air battery technologies^6^,^7^ are to become ubiquitously practical on a large scale. To date, expensive noble metals based electro-catalysts are still widely prevalent due to their excellent characteristics. These include primarily low over-potential, excellent reaction kinetics as well as outstanding long term durability in acidic media for oxygen reduction reaction (ORR) in proton exchange membrane (PEM) fuel cells (PEMFCs), oxygen evolution reaction (OER) in metal air batteries^1^ as well as hydrogen generation from solar energy and electricity driven water splitting reactions^8^. Several promising noble metal-free electro-catalysts such as Mn-oxide (MnO_2_, Mn_3_O_4_)^9^,^10^, spinels for example, NiCo_2_O_4_^11^, La-based oxide electro-catalysts (e.g. LaNiO_3_, LaCoO_3_)^1^ have all been developed albeit primarily for alkaline/neutral fuel cells and water electrolysis reactions. However, PEMFCs or PEM based water electrolysis under acidic conditions have several advantages over alkaline/neutral based systems such as higher efficiency, superior production rates, increased product purity and lastly, more compact design^12^,^13^. Widespread commercial development of PEM based systems in acidic media has been largely thwarted due to the use of expensive noble metals based electro-catalysts (Pt, IrO_2_) and hence, DOE has targeted a precious metal loading of less than or equal to 0.125 mg_noble metal_/cm^2 ^ 14.

There have been significant research efforts focused at reducing noble metal content by mixing transition metal oxides with IrO_2_ and/or RuO_2_ (e.g. Ir_1−x_Sn_x_O_2−y_F_y_, Ir_1−x_Nb_x_O_2−y_F_y_, RuO_2_–SnO_2_, IrO_2_–Ta_2_O_5_, etc.) for OER^15^,^16^. Pt-transition metal binary and ternary alloys, crystalline lattice oriented controlled alloyed structures [e.g. Pt_3_Ni(111)] and core-shell structures have been accordingly widely studied for ORR^17^,^18^. It is therefore highly important to identify and develop noble metal-free electro-catalyst devoid of any noble metal content exhibiting superior electronic conductivity, excellent charge transfer kinetics, high electrochemical active surface area (ECSA), outstanding electrochemical activity for OER and ORR, combined with superior long term electrochemical stability as well as excellent methanol tolerance for use in direct methanol fuel cell (DMFC) cathodes^2^,^3^. Thus far, no noble metal-free electro-catalyst has been identified and developed exhibiting superior electrochemical activity and stability for ORR or OER in PEMFCs or PEM based water electrolysis reactions, respectively, due to a combination of the prevalent harsh acidic operating conditions and sluggish reaction kinetics together with inferior stability of the electro-catalyst system resulting in poor electrochemical performance in comparison to the noble metals based electro-catalysts.

The present study was therefore executed primarily to identify novel noble metal-free electro-catalyst for ORR and OER in acidic media utilizing a theoretical and experimental approach. Manganese oxide (MnO_x_) is known to exhibit promising electro-catalytic activity for ORR in alkaline fuel cells and OER in water electrolysis as well as metal-air batteries and has received special attention due to its abundance, low cost, environment friendliness and good stability in alkaline media^19^,^20^. However, MnO_x_ displays poor stability in acidic media as well as low electronic conductivity limiting the desired fast charge transfer kinetics rendering it inferior as an electro-catalyst for ORR and OER in acidic media^19^. In the present work, we have for the very first time studied the copper manganese oxide based electro-catalyst system for ORR and OER in PEM based fuel cells and water electrolysis system, respectively, exploiting the cost effectiveness and environmentally benign nature of Mn. Furthermore, with the introduction of Cu into the parent Mn-oxide, a highly efficient electro-catalyst operating in acidic media was developed in contrast to the known neutral and basic media functioning Mn-oxides^21^,^22^. The first-principles calculations of the total energies and electronic structures have also been carried out to identify a suitable composition in the Cu-Mn-O based electro-catalyst system. Based on the theoretical calculations, Cu_1.5_Mn_1.5_O_4_ and x wt.% F doped Cu_1.5_Mn_1.5_O_4_ (x = 5, 10, 15), denoted as Cu_1.5_Mn_1.5_O_4_:5F, Cu_1.5_Mn_1.5_O_4_:10F and Cu_1.5_Mn_1.5_O_4_:15F in this study, respectively, have been explored as highly active and durable electro-catalyst system possessing the unique electronic structure leading to adsorption and desorption of the reaction intermediates similar to that of Pt for ORR and IrO_2_ for OER. Fluorine is used as a dopant for Cu_1.5_Mn_1.5_O_4_ to improve the electronic conductivity of Cu_1.5_Mn_1.5_O_4_, analogous to the ubiquitous use of F in transparent conductive oxides for solar cells, heat mirrors, etc, as well as the demonstrated role of F in improving the electrochemical activity of reduced noble metal containing (Ir,Sn,Nb)O_2_ system as previously reported by the present authors^16^,^23^. The synthesis of F doped Cu_1.5_Mn_1.5_O_4_ offers unique opportunity for tailoring the electronic structure, physical, electronic and electro-catalytic properties of MnO_x_ to match the characteristics of noble metal and noble metal oxide electro-catalyst systems. Thus, for the very first time, the present report documents results of theoretical first principles studies, experimental synthesis, physical characterization and electrochemical performance of nanostructured Cu_1.5_Mn_1.5_O_4_:x wt.% F (x = 0, 5, 10, 15) electro-catalysts for OER in PEM based water electrolysis and ORR in PEMFCs and DMFCs.

## Results and Discussion

For the computational study, the well-known concept proposed by J.K. Nørskov *et al*.^24^,^25^ was employed for conducting qualitative assessment of the electrochemical activity of electro-catalysts. The concept is based on the introduction of a simple descriptor for determining the surface catalytic activity of the electro-catalysts. This descriptor has been defined as a gravity center of the transition metal d-band ε_d_ usually located in the vicinity of the Fermi level^3^. An optimal position of the d-band center will thus provide an optimal interaction between the electro-catalyst surface and various species participating in the electro-catalytic reactions predominantly occurring on the surface leading to the expected maximum electrochemical activity^3^. Such an optimal interaction will thus allow the reactants and products to both adsorb on the surface and desorb most efficiently. Hence, an adjustment of the d-band center position with respect to the Fermi level will likely play a critical role in designing novel highly active electro-catalysts as discussed herein.

**Figure 1:**
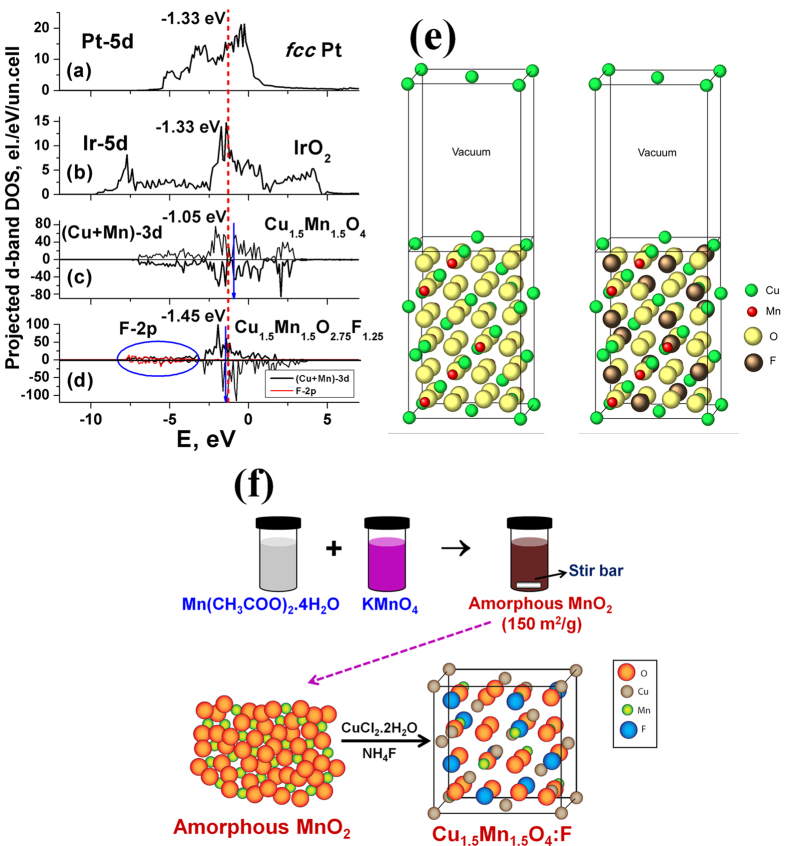
Projected d-band density of electronic states calculated for (**a**) Pt, (**b**) IrO_2_, (**c**) Cu_1.5_Mn_1.5_O_4_, and (**d**) Cu_1.5_Mn_1.5_O_2.75_F_1.25_. F-2p electronic states also shown in Fig. 1d. Fermi level is set to zero, a vertical dashed line represents d-band centers for pure Pt and IrO_2_, while arrows denote corresponding d-band centers for Cu_1.5_Mn_1.5_O_4_ and Cu_1.5_Mn_1.5_O_2.75_F_1.25_, (**e**) Slab model for Cu_1.5_Mn_1.5_O_4_, (left) and Cu_1.5_Mn_1.5_O_2.75_F_1.25_ (right) system, (**f**) Schematic illustration of the synthesis process of high surface area Cu_1.5_Mn_1.5_O_4_:F nanoparticles (NPs).

Figure 1 shows the projected d-band densities of states together with corresponding centers of these zones marked with vertical arrows on the graphs for pure Pt, IrO_2_, Cu_1.5_Mn_1.5_O_4_ and Cu_1.5_Mn_1.5_O_2.75_F_1.25_ (containing ~9.7 wt.% of F). The d-band positions of state of the art electro-catalysts Pt and IrO_2_ marked with a dashed vertical line could serve as a benchmark for the optimal electro-catalytic activity of the designed electro-catalysts. Our calculations show the same d-band center positions relative to the Fermi level of both Pt and IrO_2_ at ~(−1.33 eV) suggesting similar interaction between the electro-catalyst surface and the various intermediate species formed in both ORR and OER environments, because these reactions deal with virtually the same intermediate species, but occurring in the opposite directions. Thus, closer the corresponding d-band center of the electro-catalyst to the d-band center position of Pt or IrO_2_, the better is the expected overall electrochemical activity of the electro-catalyst as is validated and reported herein.

Calculated projected 3d-electronic density of states of Cu and Mn elements in Cu_1.5_Mn_1.5_O_4_ are shown in Fig. 1c with corresponding d-band center located at −1.05 eV *vs* Fermi level, which although is slightly above the Pt or IrO_2_ benchmark line, however is in the very vicinity, indicating the relatively high electro-catalytic activity to be expected of Cu_1.5_Mn_1.5_O_4_. An introduction of F into Cu_1.5_Mn_1.5_O_4_ shown in Fig. 1d modifies the overall electronic structure such that formation of the hybridized F2p-Mn3d electronic states below −7 eV (shown by the oval) shifts the d-band center downward to −1.45 eV which is slightly below that of Pt or IrO_2_ (−1.33 eV). Assuming linear shift of the d-band center with increase in F-content, the most optimal F-content bringing the overall d-band center of F-doped Cu_1.5_Mn_1.5_O_4_ right to the Pt or IrO_2_ benchmark position should be approximately in the range of 8–10 wt.% of F which is similar to the experimentally determined optimal F content of 10 wt.% (discussed later). Thus, F-doping in Cu_1.5_Mn_1.5_O_4_ leads to the modification of the electronic structure in general, causing a shift of the d-band center position in particular, thereby improving the overall electrochemical catalytic activity of Cu_1.5_Mn_1.5_O_4_.

**Figure 2:**
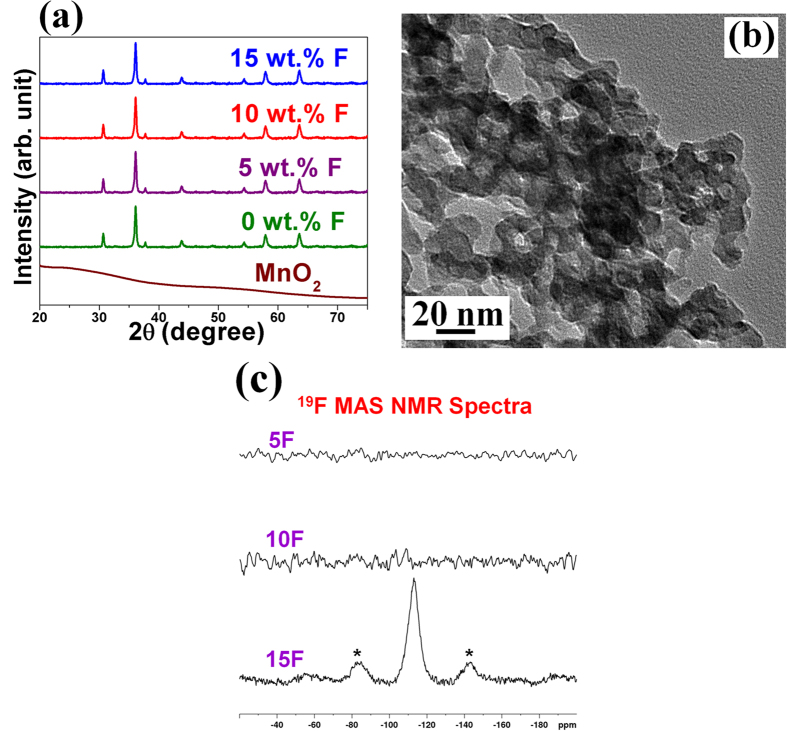
(**a**) The XRD patterns of amorphous MnO_2_ and Cu_1.5_Mn_1.5_O_4_:F of different F content in wide angle 2θ scan, (**b**) The bright field TEM image Cu_1.5_Mn_1.5_O_4_:10F shows the presence of fine particles in the nanometer range (~8–10 nm), (**c**) ^19^F MAS NMR spectra of Cu_1.5_Mn_1.5_O_4_:5F, Cu_1.5_Mn_1.5_O_4_:10F and Cu_1.5_Mn_1.5_O_4_:15F; spinning side bands are marked by asterisks.

The XRD patterns of chemically synthesized Cu_1.5_Mn_1.5_O_4_ and Cu_1.5_Mn_1.5_O_4_:F of different F content, displayed in Fig. 2a, shows the single phase cubic structure with all peaks corresponding to Cu_1.5_Mn_1.5_O_4_ (JCPDS card no: 70–0260) devoid of any secondary phase, suggesting complete incorporation of F into the lattice of Cu_1.5_Mn_1.5_O_4_. The lattice parameters of Cu_1.5_Mn_1.5_O_4_ and Cu_1.5_Mn_1.5_O_4_:F for all the compositions is *a*~0.827 nm and molar volume of ~85.16 cm^3^/mol, which is in good agreement with the reported literature value suggesting no significant effect of F doping on the molar volume of Cu_1.5_Mn_1.5_O_4_:F 26. This can possibly be due to the comparable ionic radius of O^−2^ (125 pm) and F^−1^ (120 pm)^27^. The effective crystallite size of Cu_1.5_Mn_1.5_O_4_ and Cu_1.5_Mn_1.5_O_4_:F for the different F content (calculated using the Scherrer formula) is ~8–10 nm reflecting the nano-crystalline nature of Cu_1.5_Mn_1.5_O_4_:F with negligible effect of F doping on the crystallite size of Cu_1.5_Mn_1.5_O_4_. The measured BET surface area of Cu_1.5_Mn_1.5_O_4_ and Cu_1.5_Mn_1.5_O_4_:F is ~109 m^2^/g, which further confirms that the particle size of all the synthesized compositions as discussed before (Table 1) is almost unaffected by doping of F into the oxide lattice.

The SEM micrograph combined with the EDX pattern of Cu_1.5_Mn_1.5_O_4_:10F shows clearly the presence of Cu, Mn and O (Supplementary Figs S1 and S2). The quantitative elemental composition analysis of Cu_1.5_Mn_1.5_O_4_:10F obtained by EDX confirmed that the measured elemental composition of Cu and Mn is close to the nominal composition. The elemental x-ray maps of Cu, Mn and O of Cu_1.5_Mn_1.5_O_4_:10F (Supplementary Fig. S1) also show the homogeneous distribution of Cu, Mn and O within the particles with no perceivable segregation of any of the species at any specific sites. The bright field TEM image taken for a representative composition of the oxide, Cu_1.5_Mn_1.5_O_4_:10F (Fig. 2b), shows nanometer sized particles in the size range ~8–10 nm which is in good agreement with the XRD analysis. The HRTEM image of Cu_1.5_Mn_1.5_O_4_:10F, shown in Supplementary Fig. S3, also exhibits the lattice fringes with inter-planar spacing of ~0.249 nm corresponding well with the (113) inter-planar spacing of cubic Cu_1.5_Mn_1.5_O_4_:10F determined from XRD analysis.

The oxidation states of the elements has been assessed by conducting x-ray photoelectron spectroscopy (XPS) on the electro-catalysts. The XPS spectrum of Cu in Cu_1.5_Mn_1.5_O_4_:0F shows the broad peak spanning between ~940 eV–945 eV binding energies corresponding to Cu^2+^ satellites and the peaks centered at ~931 eV and ~934 eV suggest clearly the presence of monovalent (Cu^+^) and divalent copper (Cu^2+^), respectively (Supplementary Fig. S4)^28^,^29^. The XPS spectrum of Mn (Supplementary Fig. S5) is similar to that reported earlier and indicates the presence of both Mn^3+^ and Mn^4+ ^28,29. The presence of F however, could not be unequivocally ascertained by XPS analysis similar to the observation summarized in earlier reports^16^,^30^,^31^. However, the positive shift of ~0.4 eV in Cu 2p_3/2_ and Mn 2p_3/2_ peaks of Cu_1.5_Mn_1.5_O_4_:10F observed (Supplementary Figs S4 and S5) compared to that of Cu_1.5_Mn_1.5_O_4_ is indicative of a stronger binding possibly due to the higher electro-negativity of fluorine incorporated into the lattice similar to our earlier reports serving as an indirect evidence of F incorporation into the oxide lattice^16^,^30^,^31^. Direct evidence for the presence of F has nevertheless been confirmed by solid state NMR spectroscopy. Figure 2c shows dramatic loss of the ^19^F NMR signal for Cu_1.5_Mn_1.5_O_4_:5F and Cu_1.5_Mn_1.5_O_4_:10F presumably due to the large ^19^F-electron hyperfine interactions owing to the unpaired electrons arising from the paramagnetic Cu and Mn centers, indicating the position of F atoms close to Mn/Cu in the lattice. Figure 2c and Supplementary Fig. S6 however, shows clearly the ^19^F resonances at ~(−110 ppm) for Cu_1.5_Mn_1.5_O_4_:15F and Cu_1.5_Mn_1.5_O_4_:20F samples indicating the diamagnetic nature and also suggesting the position of F atoms farther away from the metal centers. Thus, NMR results not only confirm the presence of F in Cu_1.5_Mn_1.5_O_4_:F but also provides additional information for the first time about the proximity of F to the metal centers in the oxide.

**Figure 3:**
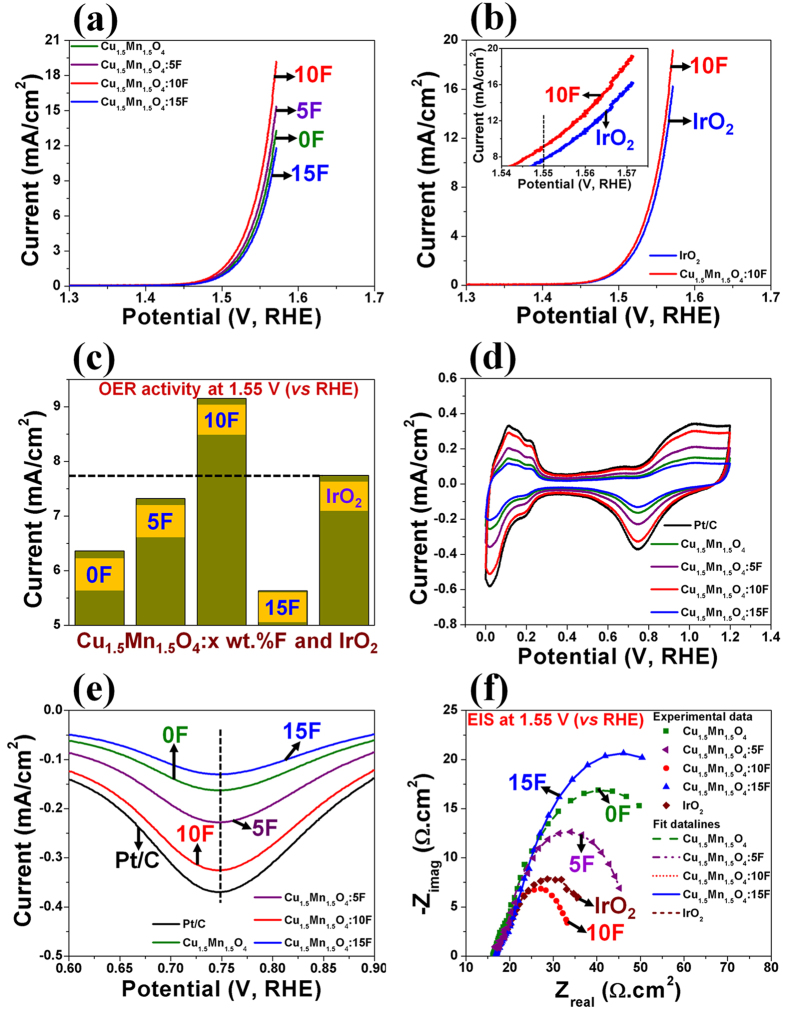
(**a**) The polarization curve of Cu_1.5_Mn_1.5_O_4_:F of different F content using total loading of 1 mg/cm^2^ obtained in 0.5 M H_2_SO_4_ solution at 40 °C with scan rate of 5 mV/sec after iR_Ω_ correction, (**b**) The polarization curve of Cu_1.5_Mn_1.5_O_4_:10F using total loading of 1 mg/cm^2^ and in-house synthesized IrO_2_ using total loading of 0.15 mg/cm^2^ obtained in 0.5 M H_2_SO_4_ solution at 40 °C with a scan rate of 5 mV/sec after iR_Ω_ correction, (**c**) Comparison of electrochemical activity for OER at ~1.55 V (*vs* RHE) between Cu_1.5_Mn_1.5_O_4_:F of different F content (total loading of 1 mg/cm^2^) and IrO_2_ (total loading of 0.15 mg/cm^2^) (**d**) The cyclic voltammograms (CVs) of Cu_1.5_Mn_1.5_O_4_:F of different F content and Pt/C, measured in N_2_ saturated 0.5 M H_2_SO_4_ at 26 °C at scan rate of 5 mV/sec using total loading of 50 μg/cm^2^ for Cu_1.5_Mn_1.5_O_4_:F and Pt loading of 30 μg_Pt_/cm^2^ for Pt/C, (**e**) The magnified view between ~0.6 V–~0.9 V (*vs* RHE) of CV curves obtained in N_2_-saturated 0.5 M H_2_SO_4_ at 26 °C at scan rate of 5 mV/sec using total loading of 50 μg/cm^2^ for Cu_1.5_Mn_1.5_O_4_:F of different F content and Pt loading of 30 μg_Pt_/cm^2^ for Pt/C, (f) EIS spectra (for OER) of Cu_1.5_Mn_1.5_O_4_:F of different F content (total loading of 1 mg/cm^2^) and in-house synthesized IrO_2_ (total loading of 0.15 mg/cm^2^) performed at ~1.55 V (*vs* RHE) in 0.5 M H_2_SO_4_ at 40 °C in the frequency range of 100 mHz to 100 kHz (using amplitude of 10 mV).

The onset potential of OER for Cu_1.5_Mn_1.5_O_4_ and Cu_1.5_Mn_1.5_O_4_:F of all compositions is ~1.43 ± 0.001 V (*vs* RHE) which is similar to that of the in-house synthesized as well as commercially obtained IrO_2_ (also reported earlier^32^) (Fig. 3a,b and Table 1). This clearly suggests similar reaction polarization^3^ of Cu_1.5_Mn_1.5_O_4_:F containing different F content to that of the in-house synthesized IrO_2_, which is in accordance with the results from the theoretical study (Fig. 1). Cu_1.5_Mn_1.5_O_4_:F with different F content also exhibit the peak potential corresponding to reduction of surface oxides of ~0.75 ± 0.001 V (*vs* RHE) similar to that of Pt/C^17^ (Fig. 3d,e). The similar peak potential corresponding to the reduction of surface oxides for Cu_1.5_Mn_1.5_O_4_:F and Pt/C suggests similar binding strength of oxygen containing species (OH, O, O_2_) on the surface of each electro-catalyst composition^33^,^34^,^35^ and thus, similar reaction polarization^3^ for ORR of Cu_1.5_Mn_1.5_O_4_:F and Pt/C, which is in agreement with the results of the theoretical study (Fig. 1). These results corroborate the unique electronic structure of Cu_1.5_Mn_1.5_O_4_:F of different F content determined by theory mimicking the electronic characteristics of the noble metal systems thus also contributing to the lower reaction polarization similar to that of the noble metal containing electro-catalysts.

The electrolyte solution resistance (R_s_), electrode resistance (R_e_) and bubble resistance (R_bub_) are mainly responsible for the linear nature of the polarization curve and non-linearity observed in the Tafel plot^36^,^37^. Thus, to study the inherent electrochemical activity of the electro-catalysts, ohmic resistance (R_Ω_) correction (iR_Ω_ = iR_s_ + iR_e_)^2^,^3^,^4^,^5^,^38^ has been conducted in the polarization and cyclic voltammogram (CV) curves. The values of R_s_ and R_e_ of the different electro-catalysts are obtained from electrochemical impedance spectroscopy (EIS) measurements (discussed later) and given in Tables 1 and 2. The current density for the in-house synthesized IrO_2_ electro-catalyst (total loading = 0.15 mg/cm^2^) is ~7.74 ± 0.0001 mA/cm^2^ at ~1.55 V (*vs* RHE, typical potential selected for assessing the electrochemical activity for OER^16^) (Fig. 3a–c and Table 1). Cu_1.5_Mn_1.5_O_4_, Cu_1.5_Mn_1.5_O_4_:5F, Cu_1.5_Mn_1.5_O_4_:10F and Cu_1.5_Mn_1.5_O_4_:15F (total loading = 1 mg/cm^2^) display excellent electro-catalytic activity for OER with current density of ~6.36 ± 0.001 mA/cm^2^, ~7.32 ± 0.001 mA/cm^2^, ~9.15 ± 0.0001 mA/cm^2^ and ~5.63 ± 0.001 mA/cm^2^ at identical potential of ~1.55 V (*vs* RHE), respectively (Table 1). Thus, Cu_1.5_Mn_1.5_O_4_, Cu_1.5_Mn_1.5_O_4_:5F, Cu_1.5_Mn_1.5_O_4_:10F and Cu_1.5_Mn_1.5_O_4_:15F show remarkable electrochemical activity, i.e., ~83%, ~95%, ~118% and ~73% of that of the in-house synthesized IrO_2_ (Fig. 3c).

**Figure 4:**
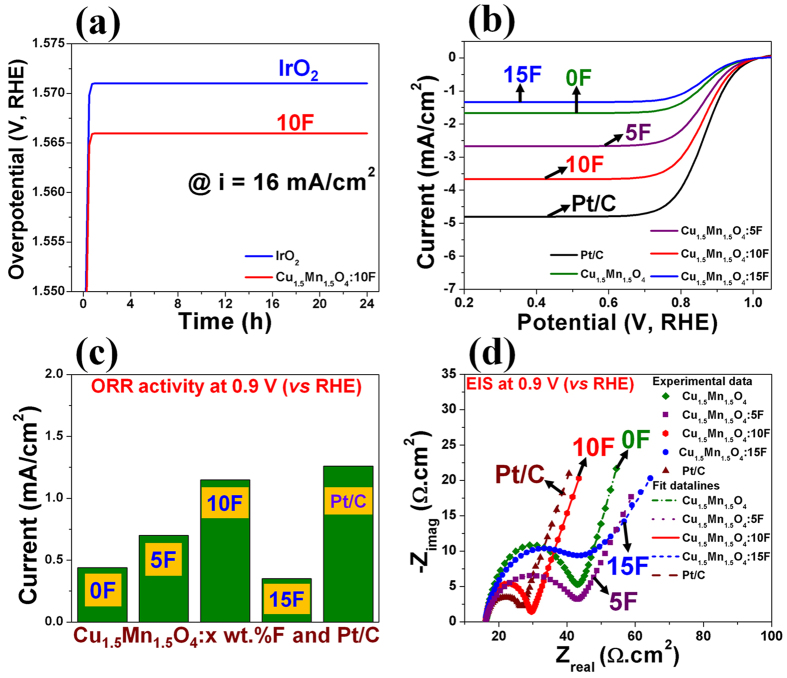
(**a**) Galvanostatic (constant current) measurement of electrochemical activity of Cu_1.5_Mn_1.5_O_4_:10F (total loading = 1 mg/cm^2^) and in-house synthesized IrO_2_ (total loading = 0.15 mg/cm^2^) performed in 0.5 M H_2_SO_4_ electrolyte solution at 40 °C at constant current of ~16 mA/cm^2^, (**b**) The polarization curves of Cu_1.5_Mn_1.5_O_4_:F of different F content and Pt/C obtained in O_2_-saturated 0.5 M H_2_SO_4_ solution at 26 °C with rotation speed of 2500 rpm and scan rate of 5 mV/sec after iR_Ω_ correction using total loading of 50 μg/cm^2^ for Cu_1.5_Mn_1.5_O_4_:F and Pt loading of 30 μg_Pt_/cm^2^ for Pt/C, (**c**) Comparison of electrochemical activity for ORR at ~0.9 V (*vs* RHE) between Cu_1.5_Mn_1.5_O_4_:F of different F content (total loading of 50 μg/cm^2^) and Pt/C (Pt loading of 30 μg_Pt_/cm^2^). (**d**) EIS spectra (for ORR) of Cu_1.5_Mn_1.5_O_4_:F of different F content (total loading of 50 μg/cm^2^) and Pt/C (Pt loading of 30 μg_Pt_/cm^2^) performed at ~0.9 V (*vs* RHE) in O_2_-saturated 0.5 M H_2_SO_4_ at 26 °C in the frequency range of 100 mHz to 100 kHz (using amplitude of 10 mV).

The reaction kinetics of the Cu_1.5_Mn_1.5_O_4_:F system was further studied by conducting EIS to determine R_s_, R_e_ and charge transfer resistance (R_ct_) (Fig. 3f). The decrease in the electrode resistance (R_e_) with increase in F content up to 10 wt.% doped in Cu_1.5_Mn_1.5_O_4_ lattice can be due to improved electronic conductivity of Cu_1.5_Mn_1.5_O_4_:F up to 10 wt.% F followed by decrease in electronic conductivity (increase in R_e_) for 15 wt.% F content (Table 1), which is in accordance with previous report^39^. R_ct_ determined from the diameter of the semi-circle in the low frequency region of the EIS plot (Fig. 3f) and the Tafel slope (Supplementary Figs S7–S10 and Table 1) decrease with increase in F content with the lowest R_ct_ and Tafel slope obtained for Cu_1.5_Mn_1.5_O_4_:10F composition (~15.15 ± 0.0001 Ω.cm^2^ and ~60 ± 0.0001 mV/dec), which clearly indicates the improvement in the reaction kinetics (decrease in activation polarization^3^) with increase in the F content to the extent of 10 wt.% F. The increase in R_ct_ and Tafel slope following continued increase in F content beyond 10 wt.% can be likely attributed to the poor reaction kinetics and also reduction in the electronic conductivity (due to increase in R_e_) (Table 1). The Tafel slope of Cu_1.5_Mn_1.5_O_4_:10F (~60 ± 0.0001 mV/dec) reflects the desired two electron pathway for OER (Table 1)^16^. It is also noteworthy to understand that the R_ct_ for Cu_1.5_Mn_1.5_O_4_:10F (~15.15 ± 0.0001 Ω.cm^2^) is almost similar to that of the in-house synthesized IrO_2_ (~17.9 ± 0.0001 Ω.cm^2^) indicating the excellent OER kinetics exhibited by Cu_1.5_Mn_1.5_O_4_:10F resulting in comparable electrochemical activity (i.e., current density) to that of IrO_2_. It is likely that further optimization of the nanoscale architectures of Cu_1.5_Mn_1.5_O_4_:F of varying compositions as well as synthesizing IrO_2_ nanoparticles with high electrochemical active surface area (by further optimization of the synthesis techniques and parameters used to generate the electro-catalyst materials) will significantly help to achieve electrochemical performance in acidic media superior to IrO_2_^40^. Furthermore, Cu_1.5_Mn_1.5_O_4_:10F matches the electro-catalytic performance of IrO_2_ by presenting comparable over-potential at a high current density of ~16 mA/cm^2^ (arbitrarily selected to normalize the electrochemical catalytic activity) (Figs 3a–c,f, 4a and Table 1). It is interesting to also note that ball milled Cu_1.5_Mn_1.5_O_4_:10F (~10 m^2^/g) also reveals identical onset potential (~1.43 V vs. RHE, Supplementary Fig. S11) as the high surface area (~109 m^2^/g) chemically synthesized doped oxide material despite the ~10 fold reduction in current density owing to the increased over-potential (see Supplementary Figs S11 and S12).

The electrochemical activity for ORR is studied by comparing the current density at ~0.9 V (*vs* RHE, the typical potential used for assessing the electrochemical activity for ORR^17^,^18^) in iR_Ω_ corrected polarization curves obtained in O_2_-saturated 0.5 M H_2_SO_4_ electrolyte solution at 26 °C (Fig. 4b,c). The fluorine doped Cu_1.5_Mn_1.5_O_4_:F system exhibit excellent electrochemical activity for ORR with an onset potential of ~1 ± 0.001 V (*vs* RHE) similar to that of Pt/C^17^ mainly due to the similar reaction polarization^3^, as discussed earlier (Figs 1, 3d,e and 4b). The current density at ~0.9 V (*vs* RHE) for Pt/C (Pt loading = 30 μg_Pt_/cm^2^) is ~1.26 ± 0.0001 mA/cm^2^ (Table 2). Cu_1.5_Mn_1.5_O_4_, Cu_1.5_Mn_1.5_O_4_:5F, Cu_1.5_Mn_1.5_O_4_:10F and Cu_1.5_Mn_1.5_O_4_:15F (total loading = 50 μg/cm^2^) exhibit current density of ~0.44 ± 0.0001 mA/cm^2^, ~0.7 ± 0.001 mA/cm^2^, ~1.15 ± 0.0001 mA/cm^2^ and ~0.35 ± 0.001 mA/cm^2^ at ~0.9 V (*vs* RHE), respectively (Fig. 4c and Table 2). Thus, Cu_1.5_Mn_1.5_O_4_, Cu_1.5_Mn_1.5_O_4_:5F, Cu_1.5_Mn_1.5_O_4_:10F and Cu_1.5_Mn_1.5_O_4_:15F exhibit ~35%, ~56%, ~92% and ~28% electrochemical activity for ORR compared to that of Pt/C, respectively (Fig. 4c).

The electrochemical activity hence, increases upon F-doping in Cu_1.5_Mn_1.5_O_4_ with increasing fluorine content to the level of 10 wt.% F primarily due to a reduction in the R_ct_ (Fig. 4d) and the Tafel slope (decrease in the activation polarization^3^) (Supplementary Fig. S13 and Table 2) with the lowest obtained for Cu_1.5_Mn_1.5_O_4_:10F [~15.6 ± 0.001 Ω.cm^2^, ~68 ± 0.001 mV/dec in low current region (LCR) and ~123 ± 0.001 mV/dec in the high current region (HCR)]. These values thus imply fast reaction kinetics for the optimal 10% F doped composition Cu_1.5_Mn_1.5_O_4_:10F with corresponding increase in R_ct_ and Tafel slope for continued increase in F content beyond 10 wt.% indicating decrease in the reaction kinetics following a trend similar to the well-known volcano plots. The number of electrons involved in the ORR for Cu_1.5_Mn_1.5_O_4_:10F determined from the Koutechy-Levich plot (Supplementary Figs S14 and S15) is ~3.88, suggesting the desired direct four electron pathway of ORR proffered by Cu_1.5_Mn_1.5_O_4_:10F 33. In addition, Cu_1.5_Mn_1.5_O_4_:10F exhibits excellent methanol tolerance for use as the cathode electro-catalyst in DMFCs (Supplementary section S1 and Supplementary Figs S16 and S17). As seen previously for OER, for the same reaction polarization (onset potential), chemically synthesized high surface area Cu_1.5_Mn_1.5_O_4_:10F showed ~12 fold improved ORR activity at ~0.9 V in contrast to ball-milled Cu_1.5_Mn_1.5_O_4_:10F (due to improved kinetics) and almost similar activity to Pt/C (Supplementary Fig. S18), suggesting that additional improvement in nanoscale architecture of Cu_1.5_Mn_1.5_O_4_:10F could further improve the OER and ORR activity and thus, yield even more efficient OER and ORR electrodes.

**Figure 5:**
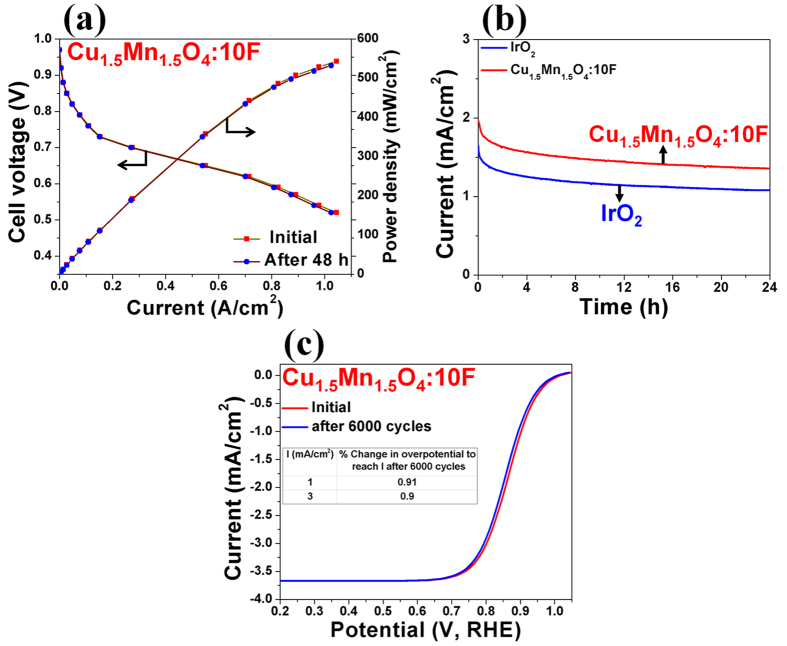
(**a**) Performance of single PEMFC full cell consisting of Cu_1.5_Mn_1.5_O_4_:10F as cathode electro-catalyst (total loading = 0.3 mg/cm^2^) and commercial 40% Pt/C (Alfa Aesar) as anode electro-catalyst (Pt loading of 0.2 mg_Pt_/cm^2^) at 80 °C and 0.1 MPa with UHP-H_2_ (200 ml/min) and UHP-O_2_ (300 ml/min) as reactant gases, (**b**) The variation of current density *vs* time in the chronoamperometry test of Cu_1.5_Mn_1.5_O_4_:10F (total loading = 1 mg/cm^2^) and in-house synthesized IrO_2_ (total loading = 0.15 mg/cm^2^), performed in 0.5 M H_2_SO_4_ solution under a constant potential of ~1.55 V (*vs* RHE) at 40 °C for 24 h, (**c**) The iR_Ω_ corrected polarization curve (initial and after 6000 cycles) of Cu_1.5_Mn_1.5_O_4_:10F (total loading = 50 μg/cm^2^) obtained after stability test in O_2_-saturated 0.5 M H_2_SO_4_ solution at 26 °C with rotation speed of 2500 rpm and scan rate of 5 mV/sec.

The polarization curve of a single PEMFC full cell fabricated by utilizing Cu_1.5_Mn_1.5_O_4_:10F (total loading = 0.3 mg/cm^2^) as the cathode electro-catalyst and commercial Pt/C (Alfa Aesar) as the anode electro-catalyst (Pt loading = 0.2 mg_Pt_/cm^2^) shows a maximum power density of ~550 mW/cm^2^ (Fig. 5a) which is ~56% of that obtained using Pt/C (Pt loading = 0.3 mg_Pt_/cm^2^) as the cathode electro-catalyst (~990 mW/cm^2^ as reported before by the present authors^3^). This is indeed a hallmark finding as the DOE recommended power density (~550 mW/cm^2^) is obtained using the novel noble metal-free electro-catalysts (Cu_1.5_Mn_1.5_O_4_:10F).

The long term electrochemical stability of the optimal composition of Cu_1.5_Mn_1.5_O_4_:10F and the in-house synthesized IrO_2_ is studied by performing chronoamperometry (CA) test wherein, the electrode was maintained at a constant voltage of ~1.55 V (*vs* RHE) in 0.5 M H_2_SO_4_ electrolyte for 24 h and the loss in electro-catalytic activity (i.e., current density) for OER has been studied (Fig. 5b). The minimal loss in current density in CA curve (similar to that of IrO_2_) combined with a minimal loss in electrochemical performance for OER in galvanostatic measurement (Fig. 4a) and even after 24 h of CA test (Supplementary Fig. S19) indicate the robustness of the composition. In addition, the measured concentration of evolved O_2_ gas during the CA test of Cu_1.5_Mn_1.5_O_4_:10F is closer or comparable to the theoretical amount of generated O_2_ suggesting ~100% Faradaic efficiency (Supplementary Fig. S20) of the electrocatalyst thus demonstrating the promise of this unique electro-catalyst system. More importantly, there is negligible loss in current density for ORR after the stability test of 6000 cycles (Fig. 5c and Supplementary Fig. S21) with concomitant absence of Cu/Mn detected in the electrolyte solution following ICP analysis post OER and ORR stability tests. Additionally, there is marginal loss in performance of single PEMFC after 48 h operation (Fig. 5a). These observations all attest to the excellent long term electrochemical stability for OER and ORR of the novel F doped composition of Cu_1.5_Mn_1.5_O_4_:10F in the highly aggressive acidic environment.

In summary, the present study demonstrates a hallmark advancement in the identification and development of novel noble metal-free electro-catalysts (Cu_1.5_Mn_1.5_O_4_:F) possessing unique electronic/molecular structure exhibiting remarkable stability and outstanding electrochemical activity for the specific composition, Cu_1.5_Mn_1.5_O_4_:10F comparable to that of IrO_2_ for OER and ~92% of that of Pt/C for ORR, the corresponding gold standard electro-catalysts, respectively. Hence, Cu_1.5_Mn_1.5_O_4_:10F indeed shows excellent promise for replacing Pt and IrO_2_ with further system modifications and optimization of the syntheses protocols and parameters. In the opinion of the authors, the system hence, represents truly a fundamental breakthrough in the pursuit of non-precious metal containing electro-catalysts for economic and efficient hydrogen generation from acid based PEM water electrolysis, while also enabling proficient power generation from fuel cells (PEMFCs, DMFCs).

## Methods

### Preparation of high surface area Cu_1.5_Mn_1.5_O_4_:x wt.% F (x = 0, 5, 10, 15) nanoparticles (NPs)

#### Synthesis of MnO_2_ NPs

Manganese acetate tetrahydrate [Mn(CH_3_COO)_2_.4H_2_O, 1.5 mmol, 99.99%, Aldrich] was dissolved in 25 mL D.I. water purified by the Milli-Q system (18 MΩ cm deionized water, Milli-Q Academic, Millipore). Separately, KMnO_4_ (1 mmol, 99.99%, Aldrich) was dissolved in 25 mL D.I. water. The KMnO_4_ solution was then added to Mn(CH_3_COO)_2_.4H_2_O solution with vigorous stirring, which immediately resulted in a brown slurry. After ~1 h stirring, the precipitate was collected by filtration and then thoroughly washed with D.I. water, followed by drying at 60 °C for 2 h which formed amorphous MnO_2_ nanoparticles with BET surface area of 150 m^2^/g (Fig. 1f).

#### Synthesis of Cu_1.5_Mn_1.5_O_4_:x wt.% F NPs

The preparation of Cu_1.5_Mn_1.5_O_4_ involved soaking as-prepared MnO_2_ NPs in stoichiometric amount of copper chloride dihydrate (CuCl_2_.2H_2_O, ≥99%, Aldrich). This was achieved by dissolving CuCl_2_.2H_2_O (3.88 mmol) in 25 ml D.I. water, followed by the addition of as-prepared MnO_2_ NPs (3.88 mmol). To generate fluorine doped oxide Cu_1.5_Mn_1.5_O_4_:F, stoichiometric ammonium fluoride (NH_4_F, 98%, Alfa Aesar) dissolved in 5 ml D.I. water was introduced in stoichiometric CuCl_2_.2H_2_O solution (20 ml) and then, stoichiometric amount of as-generated MnO_2_ NPs were added to this solution. The solution was then dried in an alumina crucible in a drying oven at 60 °C for 2 h, followed by heat treatment in air at 500 °C for 4 h (Ramp rate = 10 °C/min) in order to form Cu_1.5_Mn_1.5_O_4_:F of different F content. Cu_1.5_Mn_1.5_O_4_:x wt.% F (x = 0, 5, 10, 15) are correspondingly denoted as Cu_1.5_Mn_1.5_O_4_, Cu_1.5_Mn_1.5_O_4_:5F, Cu_1.5_Mn_1.5_O_4_:10F and Cu_1.5_Mn_1.5_O_4_:15F in this study, respectively.

### Materials Characterization

The phase analysis of the electro-catalyst materials was carried out by x-ray diffraction (XRD) using Philips XPERT PRO system employing CuK_α_ (λ = 0.15406 nm) radiation at an operating voltage and current of 45 kV and 40 mA, respectively. The XRD peak profile of Cu_1.5_Mn_1.5_O_4_:F of different F content was analyzed using the Pseudo-Voigt function to determine the Lorentzian and Gaussian contribution of the peak. The integral breadth of the Lorentzian contribution, determined from peak profile analysis using the single line approximation method after eliminating the instrumental broadening and lattice strain contribution, was used in the Scherrer formula to calculate the particle size of Cu_1.5_Mn_1.5_O_4_:F corresponding to the different compositions^2^,^3^,^41^. The lattice parameter and molar volume of the synthesized Cu_1.5_Mn_1.5_O_4_:F with different amount of fluorine have been calculated using the least square refinement techniques^2^,^3^.

Scanning electron microscopy (SEM) was conducted to investigate the microstructure of Cu_1.5_Mn_1.5_O_4_:F. Quantitative elemental analysis and distribution of elements (by elemental x-ray mapping) was obtained by utilizing the energy dispersive x-ray spectroscopy (EDX) analyzer attached with the SEM machine. Philips XL-30FEG equipped with an EDX detector system comprising of an ultrathin beryllium window and Si(Li) detector operating at 20 kV was used for the elemental and x-ray mapping analysis of the electro-catalyst compositions. Transmission electron microscopy and high resolution transmission electron microscopy (HRTEM) analysis was conducted using the JEOL JEM-2100F microscope to investigate the overall particle size and morphology of electro-catalyst materials. The specific surface area (SSA) of the electro-catalyst materials was determined by conducting nitrogen adsorption-desorption studies and analyzing the data using the Brunauer-Emmett-Teller (BET) isotherms. The powder was first vacuum degassed and then tested using a Micromeritics ASAP 2020 instrument. Multipoint BET specific surface areas have been conducted and reported for the synthesized electro-catalyst powders.

X-ray photoelectron spectroscopy (XPS) was used to investigate the valence states of Cu and Mn ions of the F doped oxides, Cu_1.5_Mn_1.5_O_4_:F. XPS analysis was carried out using a Physical Electronics (PHI) model 32–096 X-ray source control and a 22–040 power supply interfaced to a model 04–548 x-ray source with an Omni Focus III spherical capacitance analyzer (SCA). The system is routinely operated within the pressure range of 10^−8^ to 10^−9^ Torr (1.3 × 10^−6^ to 1.3 × 10^−7^ Pa). The system was calibrated in accordance with the manufacturer’s procedures utilizing the photoemission lines E_b_ of Cu 2p_3/2_ (932.7 eV), E_b_ of Au 4f_7/2_ (84 eV) and E_b_ of Ag 3d_5/2_ (368.3 eV) for a magnesium anode. All the reported intensities were obtained by dividing the experimentally determined peak areas by the instrumental sensitivity factors. Charge correction was obtained by referencing the adventitious C 1s peak to 284.8 eV. The presence of F in the synthesized electro-catalyst materials was confirmed by collecting ^19^F NMR spectra on an Avance 500 MHz Wide Bore NMR spectrometer using a 3.2 mm CP-MAS probe at a spinning speed of 14 kHz.

### Electrochemical characterization as OER electro-catalyst

Electrochemical characterization of Cu_1.5_Mn_1.5_O_4_:x wt.% F (x = 0, 5, 10, 15) NPs was performed at 40 °C (using a Fisher Scientific 910 Isotemp refrigerator circulator) on a VersaSTAT 3 (Princeton Applied Research) electrochemical workstation using a three electrode configuration in the electrolyte solution of 0.5 M sulfuric acid (H_2_SO_4_) which also served as the proton source. Prior to electrochemical testing, oxygen from the electrolyte solution was expelled by purging the electrolyte solution with ultra high pure (UHP) N_2_ gas. The electro-catalyst ink was prepared using 85 wt.% electro-catalyst and 15 wt.% Nafion 117 (5 wt.% solution in lower aliphatic alcohols, Aldrich) and further sonicated. The working electrodes were prepared by spreading the electro-catalyst ink of Cu_1.5_Mn_1.5_O_4_:x wt.% F (x = 0, 5, 10, 15) on porous Ti foil (Alfa Aesar) with the total loading of 1 mg on 1 cm^2^ area. A Pt wire (Alfa Aesar, 0.25 mm thick, 99.95%) was used as the counter electrode and mercury/mercurous sulfate (Hg/Hg_2_SO_4_) electrode (XR-200, Hach) that has a potential of +0.65 V with respect to normal hydrogen electrode (NHE) was used as the reference electrode.

The electrochemical performance of Cu_1.5_Mn_1.5_O_4_:F for OER is compared with the state of the art IrO_2_ electro-catalyst generated in-house in this study due to most commercially obtained IrO_2_ powders inevitably containing trace amounts of Ir (which is not stable under the harsh acidic operating conditions of OER^32^). Hence, the electrochemical performance of in-house synthesized IrO_2_ electro-catalyst^42^ was analyzed with total loading of 0.15 mg on 1 cm^2^ area under identical operating conditions. All the potential values reported in this study are determined with respect to reversible hydrogen electrode (RHE), calculated from the formula^5^:


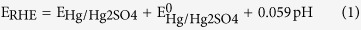


E_RHE_ is the potential versus RHE. E_Hg/Hg2SO4_ is the potential measured against the Hg/Hg_2_SO_4_ reference electrode. 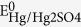 is the standard electrode potential of Hg/Hg_2_SO_4_ reference electrode (+0.65 V *vs* NHE).

The electrochemical activity of electro-catalysts for OER was determined by conducting polarization measurements in 0.5 M H_2_SO_4_ electrolyte solution employing a scan rate of 5 mV/sec at 40 °C. Polarization curves of different electro-catalysts were iR_Ω_ corrected (R_Ω_, the ohmic resistance was determined from electrochemical impedance spectroscopy analysis discussed later). The current density at ~1.55 V (*vs* RHE, which is typical potential selected for comparison of electrochemical activity of electro-catalyst for OER^16^) in iR_Ω_ corrected polarization curves was used for comparison of electrochemical performance of the different electro-catalysts. The Tafel plot after iR_Ω_ correction given by the equation η = a + b log i (plot of overpotential η *vs* log current, log i) was used to determine Tafel slope (b), which was further used to study the reaction kinetics for all the synthesized electro-catalysts.

#### Electrochemical impedance spectroscopy

The ohmic resistance (R_Ω_) (which includes resistance from components such as the electrolyte and electrode) and the charge transfer resistance (R_ct_) of electro-catalysts were determined from electrochemical impedance spectroscopy (EIS)^2^,^3^. The frequency range of 100 mHz–100 kHz (Amplitude = 10 mV) was used for EIS, which was carried out using the electrochemical work station (VersaSTAT 3, Princeton Applied Research) in 0.5 M H_2_SO_4_ electrolyte solution at 40 °C at ~1.55 V (*vs* RHE which is the typical potential used for assessing the electrochemical activity of the electro-catalyst for OER^16^) using total loading of 1 mg/cm^2^ for Cu_1.5_Mn_1.5_O_4_:F containing different F content and 0.15 mg/cm^2^ for the in-house synthesized IrO_2_. Impedance data for OER has been modeled by using the ZView software from Scribner Associates employing the R_s_(R_e_Q_1_)(R_ct_Q_dl_) equivalent circuit model to determine^2^,^3^:

R_s_ = Resistance faced at high frequency due to charge transfer in electrolyte solution.

R_e_ = Resistance for electron transfer from the electrode to current collector (Ti foil).

R_ct_ = Charge transfer resistance (i.e., polarization resistance).

Q_1_ = Constant phase element.

Q_dl_ = Contribution from both double layer capacitance and pseudocapacitance.

The ohmic resistance (R_Ω_) obtained from the EIS was used for iR_Ω_ (iR_s_ + iR_e_) correction^2^,^3^ in the polarization curves of electro-catalysts.

#### Electrochemical stability test

The electrochemical stability of the Cu_1.5_Mn_1.5_O_4_:10F electro-catalyst for long term operation was studied by conducting chronoamperometry (CA) (current *vs* time) for 24 h using 0.5 M H_2_SO_4_ as the electrolyte solution at 40 °C under a constant voltage of ~1.55 V (*vs* RHE). For comparison, the CA test was also conducted employing the in-house synthesized IrO_2_ electro-catalyst. The electrolyte (H_2_SO_4_) solution collected after 24 h of CA testing of electro-catalyst material was analyzed using inductively coupled plasma optical emission spectroscopy (ICP-OES, iCAP 6500 duo Thermo Fisher) to determine the concentration of elements leached out into the electrolyte solution from the electrode. This is extremely important since the concentration of elements in the electrolyte solution can be correlated to the electrochemical stability of electro-catalyst^2^,^3^. The concentration of the evolved O_2_ gas was also measured (after 1 h interval for 6 h) during the CA test by using a gas chromatograph utilizing Helium as the carrier gas (Agilent 7820A). Additionally, the theoretical concentration of the generated O_2_ gas is calculated from the measured current density using the Faraday’s law as under^5^,^38^:


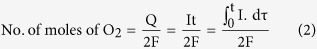


where, I is the current density, t is the time, F is the Faraday constant (96484.34 C mol^−1^) and Q is the quantity of charge in Coulomb. Galvanostatic measurements were performed for Cu_1.5_Mn_1.5_O_4_:10F and IrO_2_ in 0.5 M H_2_SO_4_ electrolyte solution at 40 °C under a constant current of ~16 mA/cm^2^ for 24 h.

### Electrochemical characterization as ORR electro-catalyst

The electrochemical characterization was carried out using a rotating disk electrode (RDE) setup. The electro-catalyst ink (85 wt.% electro-catalyst and 15 wt.% Nafion 117) was sonicated and applied to a glassy carbon (GC) disk (geometric area = 0.19 cm^2^). After solvent evaporation, the GC surface had a thin layer of electro-catalyst, which served as the working electrode. The total loading of Cu_1.5_Mn_1.5_O_4_:x wt.% F (x = 0, 5, 10, 15) was 50 μg/cm^2^. The electrochemical performance of Cu_1.5_Mn_1.5_O_4_:F for ORR is compared with the state of the art Pt/C electro-catalyst in this study. Hence, the electrochemical performance of commercially obtained 40% Pt/C electro-catalyst (Alfa Aesar) was analyzed with a Pt loading of 30 μg_Pt_ on 1 cm^2^ area tested under identical operating conditions. A Pt wire (Alfa Aesar, 0.25 mm thick, 99.95%) was used as the counter electrode and Hg/Hg_2_SO_4_ was used as the reference electrode (+0.65 V *vs* NHE).

Electrochemical characterization was conducted in an electrochemical workstation (VersaSTAT 3, Princeton Applied Research) using a three electrode cell configuration at 26 °C (using a Fisher Scientific 910 Isotemp refrigerator circulator). The cyclic voltammetry assessment was conducted in N_2_-saturated 0.5 M H_2_SO_4_ electrolyte solution by scanning the potential between ~0 V (*vs* RHE) and ~1.23 V (*vs* RHE) at a scan rate of 5 mV/sec. ORR measurement was carried out by performing polarization studies in O_2_-saturated 0.5 M H_2_SO_4_ electrolyte solution at 26 °C using the rotation speed of 2500 rpm and scan rate of 5 mV/sec. Polarization was conducted in multiple small potential steps on the RDE to reduce the contribution by the charging current and the current measurement was performed at the end of each step^3^. The current density at ~0.9 V (*vs* RHE, the typical potential for assessing electrochemical activity of electro-catalysts for ORR^17^,^18^) in iR_Ω_ corrected (R_Ω_, the ohmic resistance was determined from electrochemical impedance spectroscopy analysis described below) polarization curves of electro-catalysts was used to compare the electrochemical performance of the different electro-catalyst materials. The Tafel plot after iR_Ω_ correction given by the equation *η* = a + b log *i* (plot of overpotential *η vs* log current, log *i*) and the corresponding Tafel slope (b) has been used to study the reaction kinetics of ORR. The Koutechy-Levich equation was also used to determine the number of electrons (n) involved in the reaction^3^.


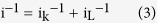



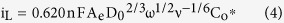


Here, i_L_ is the limiting current (A, Ampere) at ~0.6 V (*vs* RHE), i_k_ is the kinetic current (A, Ampere) observed in the absence of any mass transfer limitation, F is Faraday constant (96489 C/mol), A_e_ is the geometric area of electrode (0.19 cm^2^), D_0_ is diffusivity of O_2_ in 0.5 M H_2_SO_4_ solution (2.2×10^−5^ cm^2^/sec), ω is rotation speed (rad/sec), ν is the kinematic viscosity of water (0.01 cm^2^/sec) and C_o_^*^ is the saturated concentration of O_2_ in 0.5 M H_2_SO_4_ solution (0.25×10^−6^ mol/cm^3^)^43^,^44^.

#### Electrochemical impedance spectroscopy

Electrochemical impedance spectroscopy (EIS) was carried out to determine the ohmic resistance (R_Ω_) (which includes the resistance of various components including electrolyte and electrode) and charge transfer resistance (or polarization resistance) (R_ct_) of electro-catalysts^2^,^3^. EIS has been conducted in the frequency range of 100 mHz–100 kHz (Amplitude = 10 mV) at ~0.9 V (*vs* RHE which is typical potential for assessing electro-catalyst activity for ORR^17^,^18^) in O_2_-saturated 0.5 M H_2_SO_4_ solution at 26 °C using the electrochemical work station (VersaSTAT 3, Princeton Applied Research). The experimentally obtained EIS plot was fitted using the ZView software from Scribner Associates with an equivalent circuit model R_Ω_(R_ct_Q_1_W_o_), where Q_1_ is constant phase element and W_o_ is open circuit terminus Warburg element^3^. R_Ω_ was used for ohmic loss correction (iR_Ω_) in the polarization curves of the electro-catalysts^2^,^3^.

#### Methanol tolerance test

Methanol tolerance test was carried out for the electro-catalyst by performing polarization measurements in O_2_-saturated 0.5 M H_2_SO_4_ electrolyte solution in the presence of 1 M methanol at a rotation speed of 2500 rpm and scan rate of 5 mV/sec at 26 °C.

#### Electrochemical stability/durability test

The electrochemical stability/durability of electro-catalyst for long term operation was studied by performing cyclic voltammetry by scanning potential between ~0.6 V (*vs* RHE) and ~1.23 V (*vs* RHE) in N_2_-saturated 0.5 M H_2_SO_4_ electrolyte solution at 26 °C at scan rate of 5 mV/sec for 6000 cycles, followed by conducting polarization in O_2_-saturated 0.5 M H_2_SO_4_ solution after 6000 cycles at 26 °C using rotation speed of 2500 rpm and scan rate of 5 mV/sec^17^. Elemental analysis of the electrolyte was performed after 6000 cycles by inductively coupled plasma optical emission spectroscopy (ICP-OES, iCAP 6500 duo Thermo Fisher) to determine the amount of elements leached out into the electrolyte solution from the electrode providing vital information about the electrochemical stability of the electro-catalyst^2^,^3^.

#### Membrane electrode assembly (MEA) preparation and single PEMFC full cell test analysis

The anode and cathode electro-catalyst ink was prepared consisting of 85 wt.% electro-catalyst and 15 wt.% Nafion 117 solution (5 wt.% solution in lower aliphatic alcohols, Sigma-Aldrich). For the anode, Pt loading of commercially obtained 40% Pt/C (Alfa Aesar) electro-catalyst was 0.2 mg_Pt_/cm^2^. For the cathode, the total loading of 0.3 mg/cm^2^ was used for the Cu_1.5_Mn_1.5_O_4_:10F electro-catalyst. For comparison, 40% Pt/C (Alfa Aesar) was also studied as the cathode electro-catalyst in the single PEMFC full cell test using Pt loading of 0.3 mg_Pt_/cm^2^. The electrodes were prepared by spreading the electro-catalyst ink on teflonized carbon paper. For the single full cell testing^2^,^3^, a membrane electrode assembly was fabricated by using a Nafion 115 membrane which was sandwiched between the anode and cathode. The Nafion 115 membrane was pretreated first with 3 wt.% hydrogen peroxide solution to its boiling point to oxidize any organic impurities. Subsequently, it was boiled in D.I. water followed by immersion in boiling 0.5 M sulfuric acid solution to eliminate impurities. Finally, it was washed multiple times in D.I. water to remove any traces of remnant acid. This membrane was then stored in D.I. water to avoid dehydration. The sandwiching of Nafion 115 membrane between the anode and cathode was carried out by hot-pressing in a 25T hydraulic lamination hot press with a dual temperature controller (MTI Corporation) at a temperature of 125 °C and pressure of 40 atmosphere applied for 30 sec to ensure good contact between the electrodes and the membrane. This MEA was then used in the single cell test analysis, carried out for 48 h using the PEMFC fuel cell test set up obtained from Electrochem Incorporation at 80 °C and 0.1 MPa with UHP-H_2_ (200 ml/min) and UHP-O_2_ (300 ml/min) as reactant gases^3^.

### Computational methodology

The overall electro-catalytic activity of the electro-catalyst is expected to depend mainly on its electronic structure. The effect of compositions on the electronic structure and the electro-catalytic activity of the electro-catalyst can be well-understood from theoretical considerations. The computational component of the present study is essentially to investigate the electronic structure of pure Cu_1.5_Mn_1.5_O_4_ and F-doped Cu_1.5_Mn_1.5_O_4_. The total energy, electronic and optimized crystal structures as well as total and projected densities of electronic states for pure and F-doped Cu_1.5_Mn_1.5_O_4_ have thus been calculated using the first principles approach within the density functional theory. The electronic structure of the stable surfaces for all the electro-catalysts have been calculated in this study and the positions of corresponding d-band centers have been obtained as a first moment of *n*_*d*_(*E*): 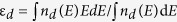, where *n*_*d*_(*E*) is the projected d-band density of states of the corresponding electro-catalyst materials. For comparative purpose, pure platinum used as the accepted gold standard electro-catalyst for ORR in fuel cells (PEMFCs, DMFCs) as well as IrO_2_ widely used as the accepted standard catalyst for OER in PEM water electrolysis have also been considered in the present study.

For calculating the total energies, electronic structure and density of electronic states, the Vienna Ab-initio Simulation Package (VASP) was used within the projector-augmented wave (PAW) method^45^,^46^,^47^ and the spin-polarized generalized gradient approximation (GGA) for the exchange-correlation energy functional in a form suggested by Perdew and Wang^48^. This program calculates the electronic structure and *via* the Hellmann-Feynman theorem, the inter-atomic forces were determined from first-principles. Standard PAW potentials were employed for the Cu, Mn, O, F, Pt and Ir potentials containing eleven, seven, six, seven, ten, and nine valence electrons, respectively. Cu_1.5_Mn_1.5_O_4_ at room temperature adopts a complex cubic crystal structure with P4_1_32 symmetry and space group # 213^49^. The bulk elementary unit cell contains 56 atoms corresponding to 8 formula units. All the surface calculations for pure and F-doped oxides have been conducted for (100) surface with thirteen atomic layer slab separated by its image in the [100] direction by a vacuum layer. Both the slab and vacuum layers have the same thickness of ~12.5 Å. In the case of F-doped oxide, 15 F-atoms have been randomly distributed over the six 8-atomic oxygen layers in the slab, thus representing the Cu_1.5_Mn_1.5_O_2.75_F_1.25_ composition corresponding to ~9.7 wt.% of F. Also, a (111) *fcc* surface calculation for pure Pt and (110) rutile type surface calculation for IrO_2_ has been conducted to achieve effective comparison. Figure 1e shows the slab model for both, the Cu_1.5_Mn_1.5_O_4_ and Cu_1.5_Mn_1.5_O_2.75_F_1.25_ compositions.

For all the electro-catalysts considered in the present study, the plane wave cutoff energy of 520 eV has been chosen to maintain a high accuracy of the total energy calculations. The lattice parameters and internal positions of atoms were fully optimized employing the double relaxation procedure and consequently, the minima of the total energies with respect to the lattice parameters and internal ionic positions have been determined. This geometry optimization was obtained by minimizing the Hellman–Feynman forces via a conjugate gradient method, so that the net forces applied on every ion in the lattice are close to zero. The total electronic energies were converged within 10^−5^ eV/un.cell resulting in the residual force components on each atom to be lower than 0.01 eV/Å/atom, thus allowing for an accurate determination of the internal structural parameters for the oxide. The Monkhorst-Pack scheme was used to sample the Brillouin Zone (BZ) and generate the *k*-point grid for all the materials considered in the present study. A choice of the appropriate number of *k*-points in the irreducible part of the BZ was considered based on the convergence of the total energy to 0.1 meV/atom.

## Additional Information

**How to cite this article**: Patel, P. P. *et al*. Noble metal-free bifunctional oxygen evolution and oxygen reduction acidic media electro-catalysts. *Sci. Rep.* **6**, 28367; doi: 10.1038/srep28367 (2016).

## Supplementary Material

Supplementary Information

## Figures and Tables

**Table 1:** Results of electrochemical characterization for OER.

Electro-catalyst	BET surface area (m^2^/g)	Onset potential for OER (V *vs* RHE)	Current density for OER at 1.55 V (*vs* RHE) (mA/cm^2^)	R_s_ (Ω.cm^2^)	R_e_ (Ω.cm^2^)	R_ct_ (Ω.cm^2^)	Tafel slope (mV/dec)
Cu_1.5_Mn_1.5_O_4_	109	1.43 ± 0.001	6.36 ± 0.001	16.38 ± 0.001	5 ± 0.001	44.9 ± 0.001	66.8 ± 0.0001
Cu_1.5_Mn_1.5_O_4_:5F	109	1.43 ± 0.001	7.32 ± 0.001	16.31 ± 0.001	4.71 ± 0.001	29.2 ± 0.001	65.7 ± 0.0001
Cu_1.5_Mn_1.5_O_4_:10F	109	1.43 ± 0.0001	9.15 ± 0.0001	16.35 ± 0.0001	3.5 ± 0.0001	15.15 ± 0.0001	60 ± 0.0001
Cu_1.5_Mn_1.5_O_4_:15F	109	1.43 ± 0.001	5.63 ± 0.001	16.36 ± 0.001	4.95 ± 0.001	47.2 ± 0.001	69.1 ± 0.0001
IrO_2_	191	1.43 ± 0.0001	7.74 ± 0.0001	16.35 ± 0.0001	3.65 ± 0.0001	17.9 ± 0.0001	–

**Table 2:** Results of electrochemical characterization for ORR.

Electro-catalyst	Current density for ORR at 0.9 V (*vs* RHE) (mA/cm^2^)	R_Ω_ (Ω.cm^2^)	R_ct_ (Ω.cm^2^)	Tafel slope (mV/dec)	
				in LCR	in HCR
Cu_1.5_Mn_1.5_O_4_	0.44 ± 0.0001	16.5 ± 0.001	31.5 ± 0.001	75 ± 0.001	130 ± 0.001
Cu_1.5_Mn_1.5_O_4_:5F	0.7 ± 0.001	16.45 ± 0.001	28.55 ± 0.001	72 ± 0.001	127 ± 0.001
Cu_1.5_Mn_1.5_O_4_:10F	1.15 ± 0.0001	16.4 ± 0.001	15.6 ± 0.001	68 ± 0.001	123 ± 0.001
Cu_1.5_Mn_1.5_O_4_:15F	0.35 ± 0.001	16.38 ± 0.001	41.62 ± 0.001	79 ± 0.001	141 ± 0.001
Pt/C	1.26 ± 0.0001	16.39 ± 0.001	9.2 ± 0.001	–	–
