# Supplementary material for: Noble metal-free bifunctional oxygen evolution and oxygen reduction acidic media electro-catalysts
Source: Sci Rep. 2016 Jul 6;6:28367. doi: 10.1038/srep28367 (PMC4933978; doi:10.1038/srep28367)
Supplement: Supplementary Information [file srep28367-s1.pdf]

Supplementary information

Noble metal-free bifunctional oxygen evolution and oxygen reduction acidic media electro-catalysts

Prasad Prakash Patel[1], Moni Kanchan Datta[2,3], Oleg I. Velikokhatnyi[2,3], Ramalinga Kuruba[2], Krishnan Damodaran[4], Prashanth Jampani[2], Bharat Gattu[1], Pavithra Murugavel Shanthi[1], Sameer S. Damle[1], Prashant N. Kumta[1,2,3,5,6*]

[1]Department of Chemical and Petroleum Engineering, Swanson School of Engineering, University of Pittsburgh, Pittsburgh, PA 15261, USA.

[2]Department of Bioengineering, Swanson School of Engineering, University of Pittsburgh, Pittsburgh, PA 15261, USA.

[3]Center for Complex Engineered Multifunctional Materials, University of Pittsburgh, PA 15261, USA.

[4]Department of Chemistry, University of Pittsburgh, PA 15260

[5]Mechanical Engineering and Materials Science, Swanson School of Engineering, University of Pittsburgh, Pittsburgh, PA 15261, USA.

[6]School of Dental Medicine, University of Pittsburgh, PA 15217, USA.

[*]Corresponding author: Prof. Prashant N. Kumta (pkumta@pitt.edu)

Department of Bioengineering, 815C Benedum Hall, 3700 O'Hara Street, Pittsburgh, PA 15261.

Tel: +1-412-648-0223, Fax: +1-412-624-3699

Table of contents:

Section S1: Methanol tolerance test of $Cu_{1.5}Mn_{1.5}O_4$:10F and Pt/C

Section S2: Synthesis of $Cu_{1.5}Mn_{1.5}O_4$:10F *via* ball-milling

Figure S1. SEM micrograph with elemental x-ray maps of $Cu_{1.5}Mn_{1.5}O_4$:10F

Figure S2. EDX spectrum of $Cu_{1.5}Mn_{1.5}O_4$:10F

Figure S3. HRTEM image showing lattice fringes with spacing of ~0.249 nm corresponding to the (113) interplanar spacing of cubic $Cu_{1.5}Mn_{1.5}O_4$:10F

Figure S4. The XPS spectra of $Cu_{1.5}Mn_{1.5}O_4$ and $Cu_{1.5}Mn_{1.5}O_4$:10F showing Cu $2p_{3/2}$ peak

Figure S5. The XPS spectra of $Cu_{1.5}Mn_{1.5}O_4$ and $Cu_{1.5}Mn_{1.5}O_4$:10F showing Mn $2p_{3/2}$ peak

Figure S6. ^{19}F MAS NMR spectra of $Cu_{1.5}Mn_{1.5}O_4$:5F, $Cu_{1.5}Mn_{1.5}O_4$:10F, $Cu_{1.5}Mn_{1.5}O_4$:15F and $Cu_{1.5}Mn_{1.5}O_4$:20F; spinning side bands are marked by asterisks

Figure S7. The Tafel plot (for OER) after iR_{Ω} correction of $Cu_{1.5}Mn_{1.5}O_4$

Figure S8. The Tafel plot (for OER) after iR_{Ω} correction of $Cu_{1.5}Mn_{1.5}O_4$:5F

Figure S9. The Tafel plot (for OER) after iR_{Ω} correction of $Cu_{1.5}Mn_{1.5}O_4$:10F

Figure S10. The Tafel plot (for OER) after iR_{Ω} correction of $Cu_{1.5}Mn_{1.5}O_4$:15F

Figure S11. The polarization curve of chemically synthesized and ball-milled $Cu_{1.5}Mn_{1.5}O_4$:10F using total loading of 1 mg/cm^2 and in-house synthesized IrO_2 using total loading of 0.15 mg/cm^2 obtained in 0.5 M H_2SO_4 solution at 40^0C with a scan rate of 5 mV/sec after iR_{Ω} correction

Figure S12. Galvanostatic (constant current) measurement of electrochemical activity of chemically synthesized and ball milled $Cu_{1.5}Mn_{1.5}O_4$:10F (total loading=1 mg/cm^2) and in-house synthesized IrO_2 (total loading=0.15 mg/cm^2) performed in 0.5 M H_2SO_4 electrolyte solution at 40^0C at a constant current of ~2 mA/cm^2

Figure S13. The Tafel plot (for ORR) after iR_Ω correction of $Cu_{1.5}Mn_{1.5}O_4$, $Cu_{1.5}Mn_{1.5}O_4$:5F, $Cu_{1.5}Mn_{1.5}O_4$:10F and $Cu_{1.5}Mn_{1.5}O_4$:15F

Figure S14. The polarization curve for ORR of $Cu_{1.5}Mn_{1.5}O_4$:10F (total loading = 50 μg/cm^2) at different rotation speeds measured in O_2-saturated 0.5 M H_2SO_4 solution at 26^0C with a scan rate of 5 mV/sec

Figure S15. The Koutechy-Levich plot for ORR of $Cu_{1.5}Mn_{1.5}O_4$:10F at ~0.6 V (*vs* RHE)

Figure S16. The polarization curve of $Cu_{1.5}Mn_{1.5}O_4$:10F obtained in O_2-saturated 0.5 M H_2SO_4 solution at 26^0C with rotation speed of 2500 rpm and scan rate of 5 mV/sec after iR_Ω correction using total loading of 50 μg/cm^2, with and without 1 M methanol in 0.5 M H_2SO_4 electrolyte solution

Figure S17. The polarization curves of Pt/C obtained in O_2-saturated 0.5 M H_2SO_4 solution at 26^0C with rotation speed of 2500 rpm and scan rate of 5 mV/sec after iR_Ω correction using Pt loading of 30 μg_{Pt}/cm^2 for Pt/C, with and without 1 M methanol in 0.5 M H_2SO_4 electrolyte solution

Figure S18. The polarization curves of chemically synthesized and ball-milled $Cu_{1.5}Mn_{1.5}O_4$:10F and Pt/C obtained in O_2-saturated 0.5 M H_2SO_4 solution at 26^0C with rotation speed of 2500 rpm and scan rate of 5 mV/sec after iR_Ω correction using total loading of 50 μg/cm^2 for $Cu_{1.5}Mn_{1.5}O_4$:10F and Pt loading of 30 μg_{Pt}/cm^2 for Pt/C

Figure S19. The iR_{Ω} corrected polarization curve of $Cu_{1.5}Mn_{1.5}O_4$:10F (total loading = 1 mg/cm^2) obtained after 24 h of chronoamperometry test in 0.5 M H_2SO_4 solution at 40^0C with a scan rate of 5 mV/sec

Figure S20. Theoretical and experimentally measured concentration of O_2 gas, measured (for 6 h) during chronoamperometry test of $Cu_{1.5}Mn_{1.5}O_4$:10F (total loading=1 mg/cm^2), performed in 0.5 M H_2SO_4 solution under a constant potential of ~1.55 V (*vs* RHE) at 40^0C

Figure S21. The cyclic voltammogram (CV) of $Cu_{1.5}Mn_{1.5}O_4$:10F measured in N_2 saturated 0.5 M H_2SO_4 at 26^0C at scan rate of 5 mV/sec using total loading of 50 $\mu g/cm^2$, initial and after 6000 cycles

Section S1: <u>Methanol tolerance test:</u>

In DMFCs, methanol cross-over from anode to cathode has detrimental effect on the fuel cell performance due to the undesired reaction with O_2 and cathode electro-catalyst.[1] Hence, methanol tolerance of $Cu_{1.5}Mn_{1.5}O_4$:10F is studied by conducting polarization studies in O_2-saturated (1 M methanol + 0.5 M H_2SO_4) electrolyte solution at 26^0C using a scan rate of 5 mV/sec and rotation speed of 2500 rpm employing a total loading of 50 $\mu g/cm^2$ for $Cu_{1.5}Mn_{1.5}O_4$:F. For comparison, methanol tolerance of commercial Pt/C is also studied with Pt loading of 30 $\mu g_{Pt}/cm^2$ under identical operating conditions. The polarization curves of $Cu_{1.5}Mn_{1.5}O_4$:10F and Pt/C with and without presence of methanol in 0.5 M H_2SO_4 electrolyte solution are shown in the **Supplementary Figs. S17-S18**, respectively. The significant increase in overpotential (~400 mV) in methanol containing electrolyte solution is observed for Pt/C (**Supplementary Fig. S18**) mainly

due to the competition between ORR and methanol electro-oxidation, which is similar to that reported earlier.[2] However, only a minimal increase in overpotential (~7 mV) is seen for $Cu_{1.5}Mn_{1.5}O_4$:10F in methanol containing electrolyte solution (**Supplementary Fig. S17**) suggesting the excellent methanol tolerance of the oxide electro-catalyst which is significantly superior to Pt/C. Hence, we believe $Cu_{1.5}Mn_{1.5}O_4$:10F is indeed a promising cathode electro-catalyst for ORR in DMFCs.

Section S2: Synthesis of $Cu_{1.5}Mn_{1.5}O_4$:10F via ball-milling:

Mixtures of CuO (Alfa Aesar, 99.5%),MnO (Alfa Aesar, 99.5%) and (NH_4F, 98%, Alfa Aesar) corresponding to the stoichiometric composition were subjected to high energy mechanical milling in a high energy shaker mill for 5 h in a stainless steel (SS) vial using 20 SS balls of 2 mm diameter with a ball to powder weight ratio 10:1. The milled powder was then heat treated in air at 500^0C for 4 h (Ramp rate=10^0C/min).

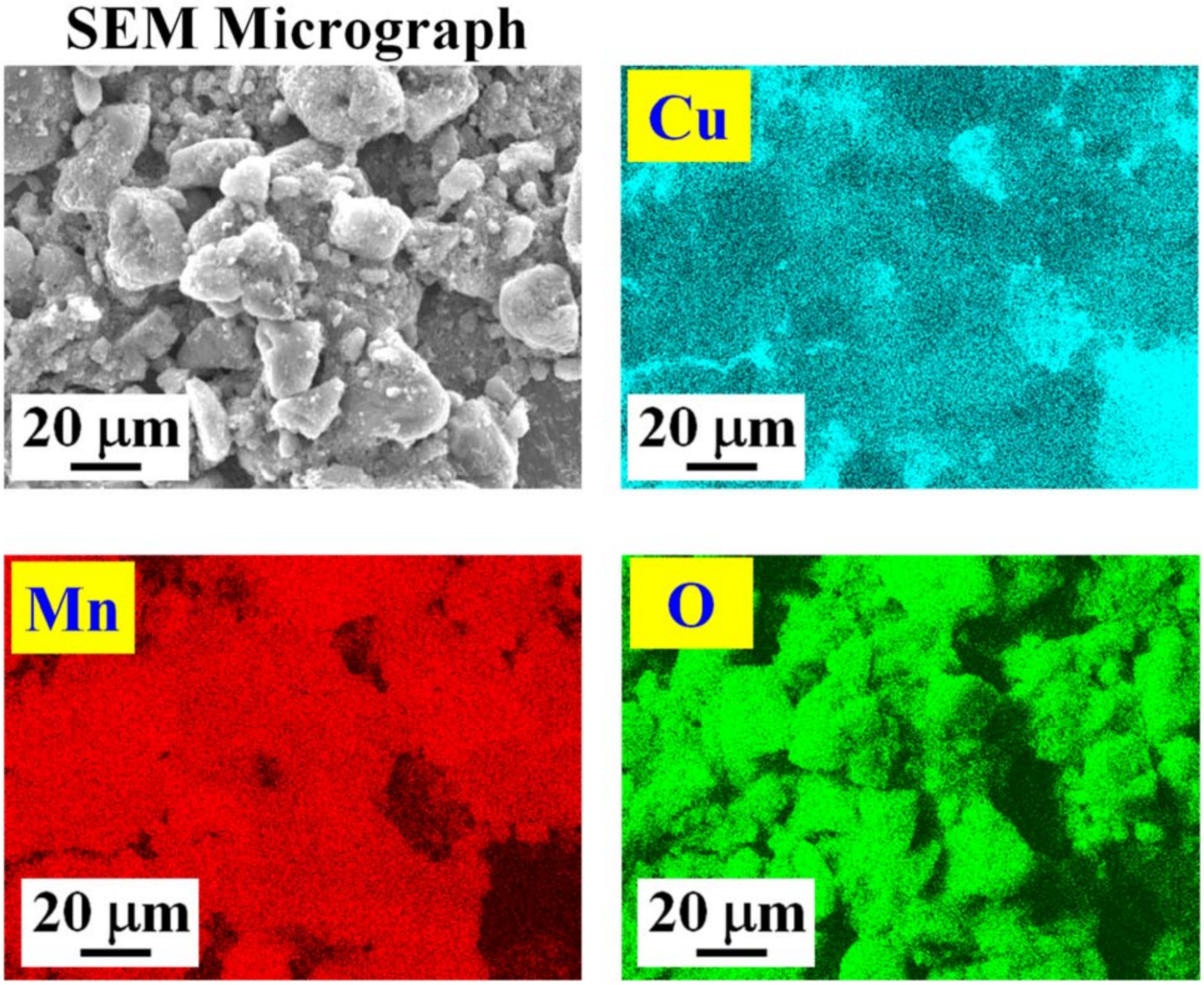

Figure S1. SEM micrograph with elemental x-ray maps of $Cu_{1.5}Mn_{1.5}O_4$:10F

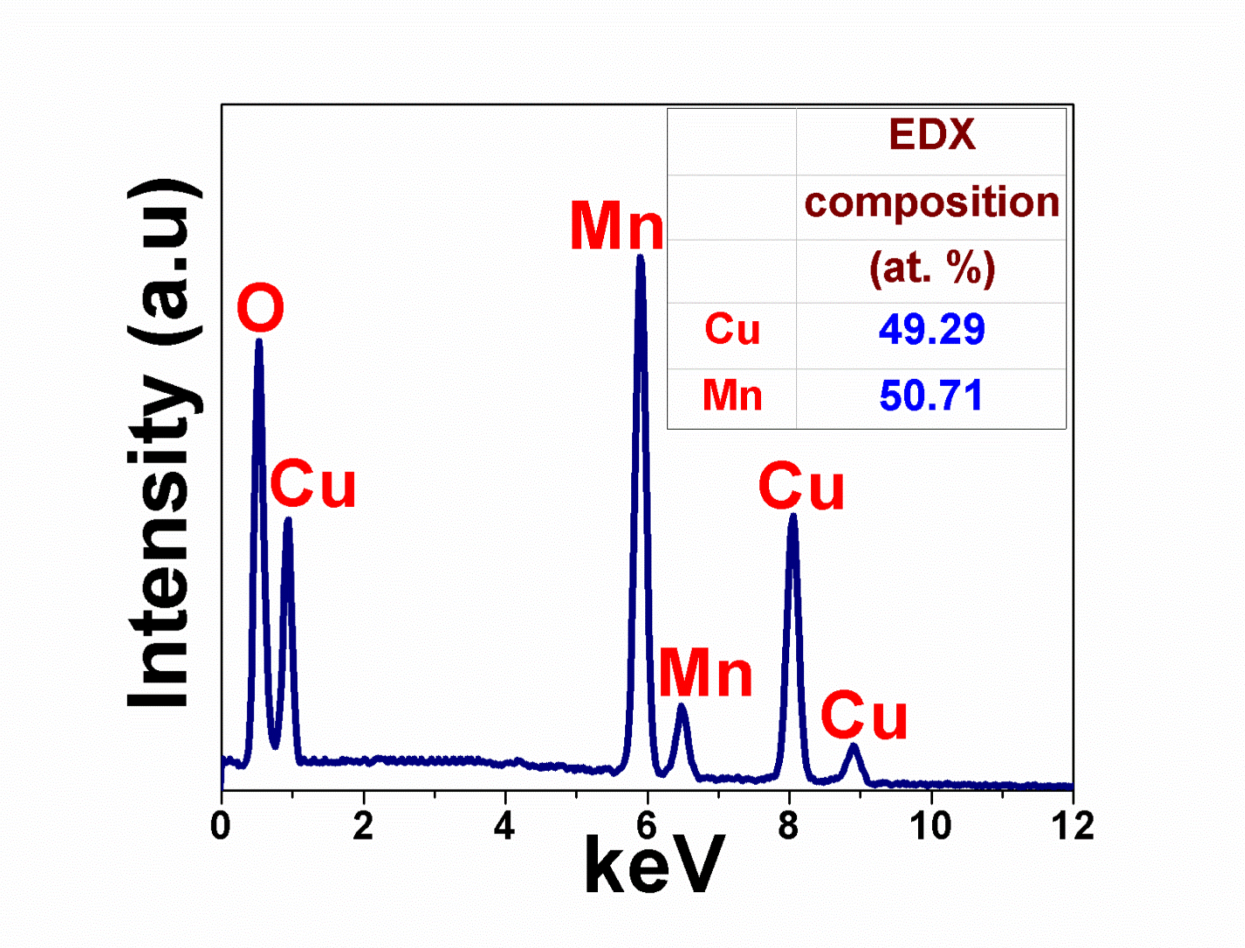

Figure S2. EDX spectrum of $Cu_{1.5}Mn_{1.5}O_4$:10F

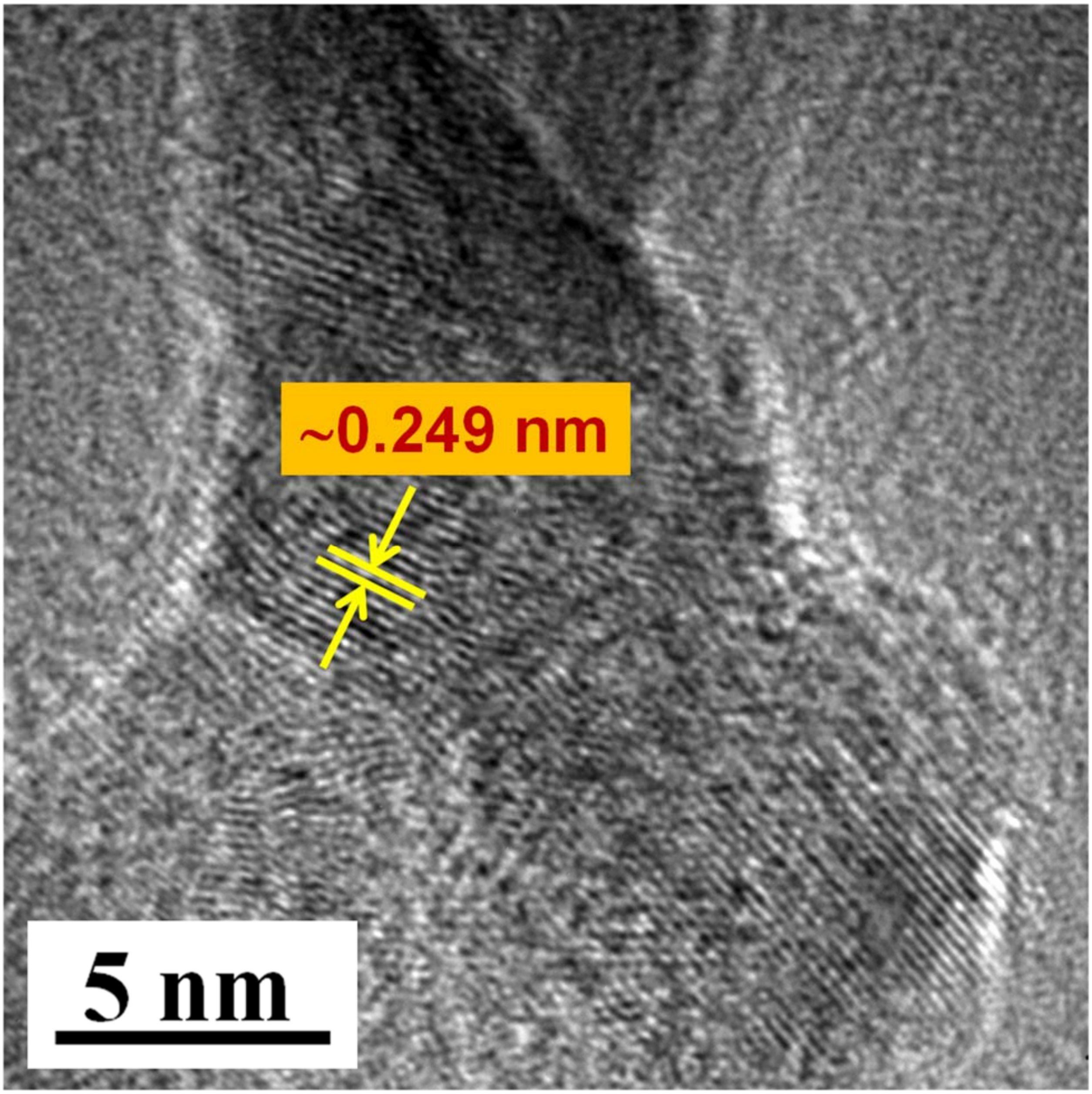

Figure S3. HRTEM image showing lattice fringes with spacing of ~0.249 nm corresponding to the (113) interplanar spacing of cubic $Cu_{1.5}Mn_{1.5}O_4$:10F

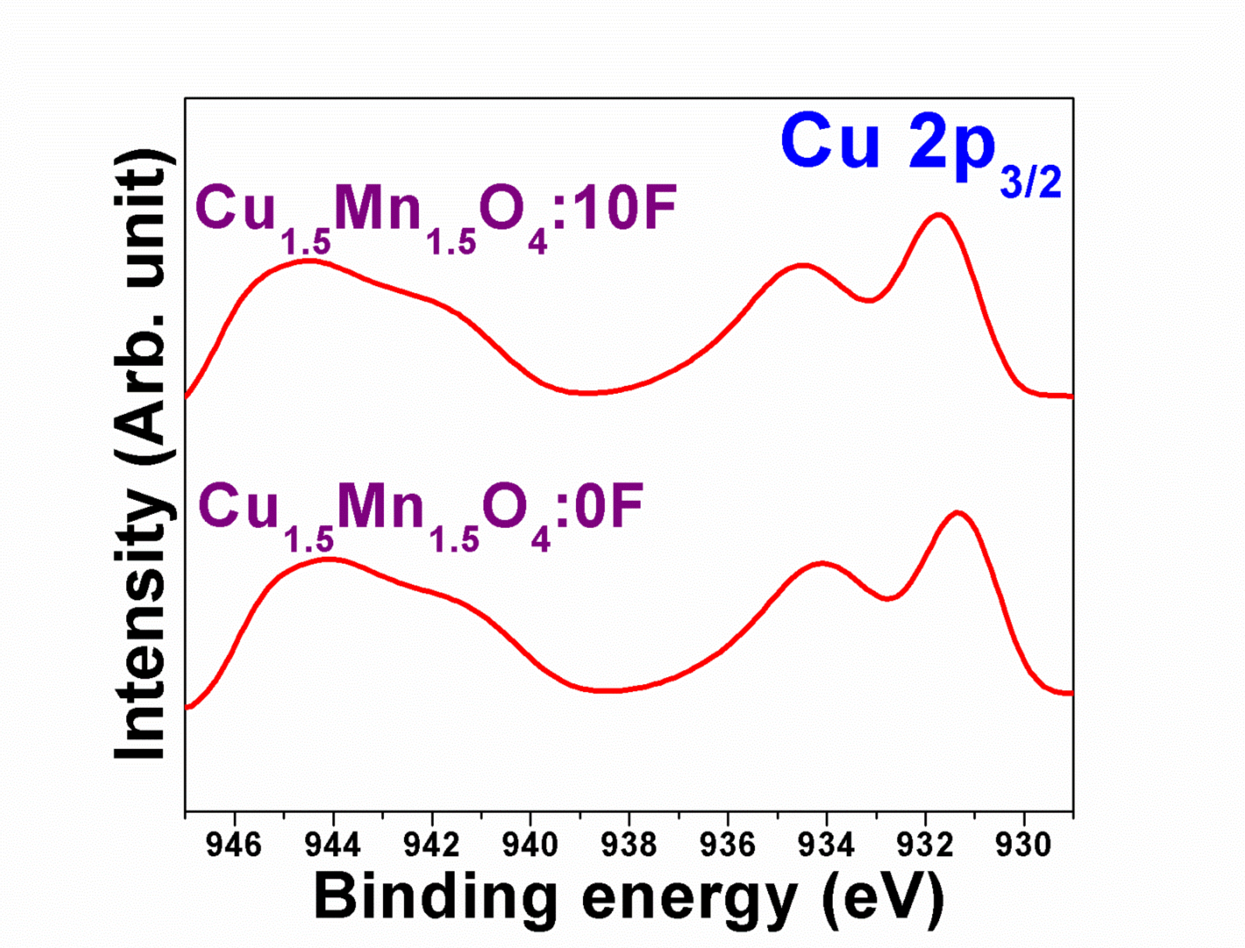

Figure S4. The XPS spectra of $Cu_{1.5}Mn_{1.5}O_4$ and $Cu_{1.5}Mn_{1.5}O_4$:10F showing Cu $2p_{3/2}$ peak

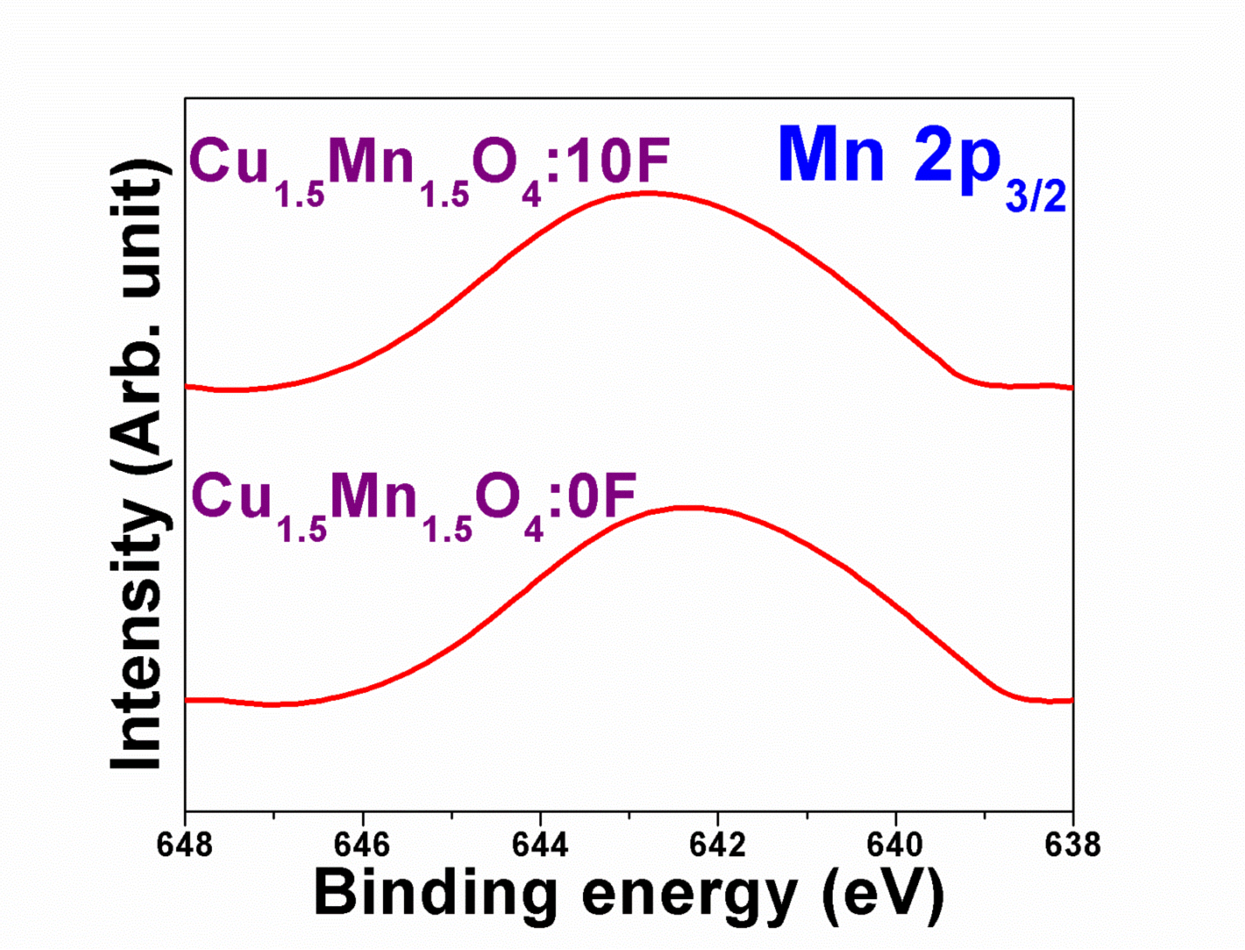

Figure S5. The XPS spectra of $Cu_{1.5}Mn_{1.5}O_4$ and $Cu_{1.5}Mn_{1.5}O_4$:10F showing Mn $2p_{3/2}$ peak

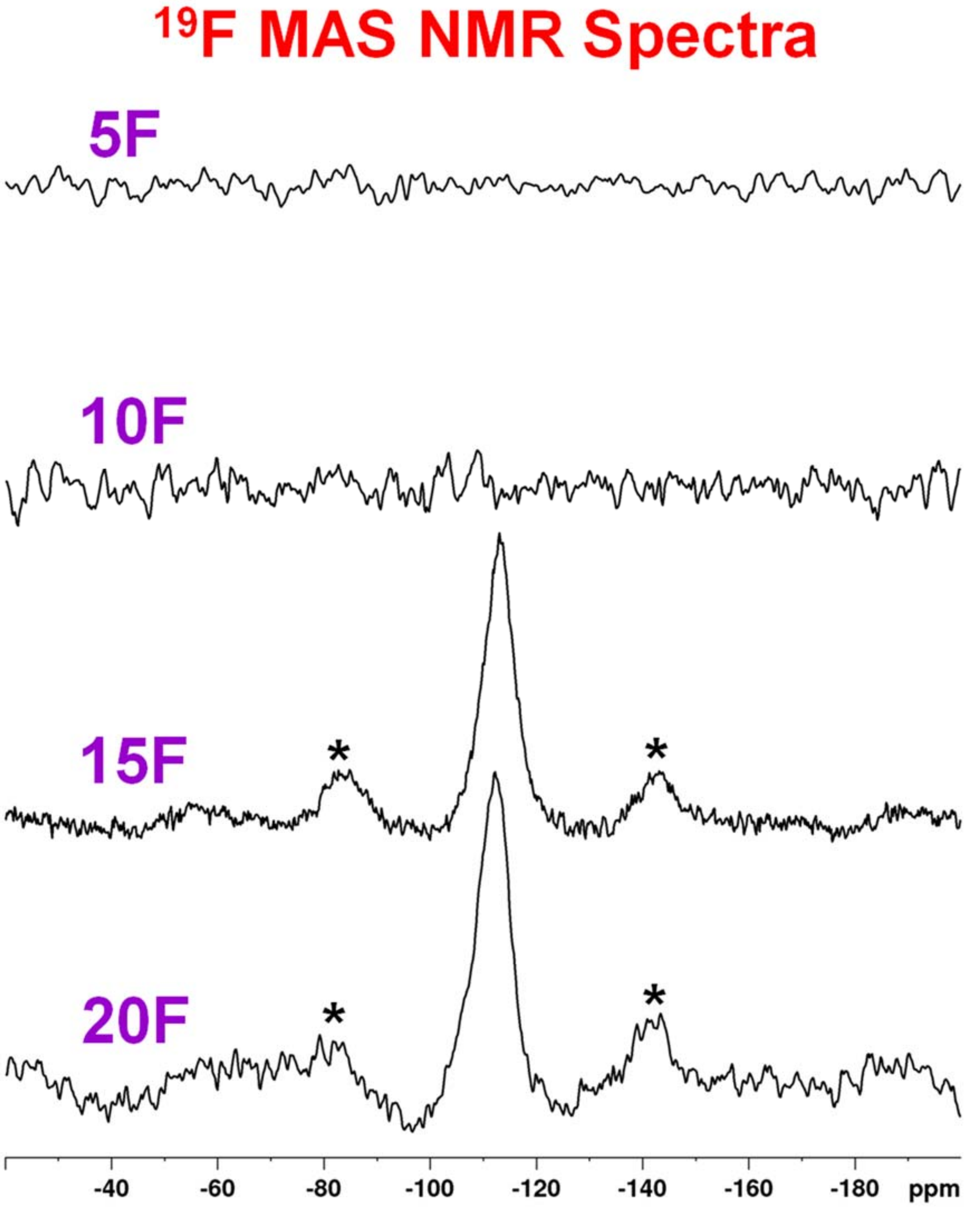

Figure S6. ^{19}F MAS NMR spectra of $Cu_{1.5}Mn_{1.5}O_4$:5F, $Cu_{1.5}Mn_{1.5}O_4$:10F, $Cu_{1.5}Mn_{1.5}O_4$:15F and $Cu_{1.5}Mn_{1.5}O_4$:20F; spinning side bands are marked by asterisks

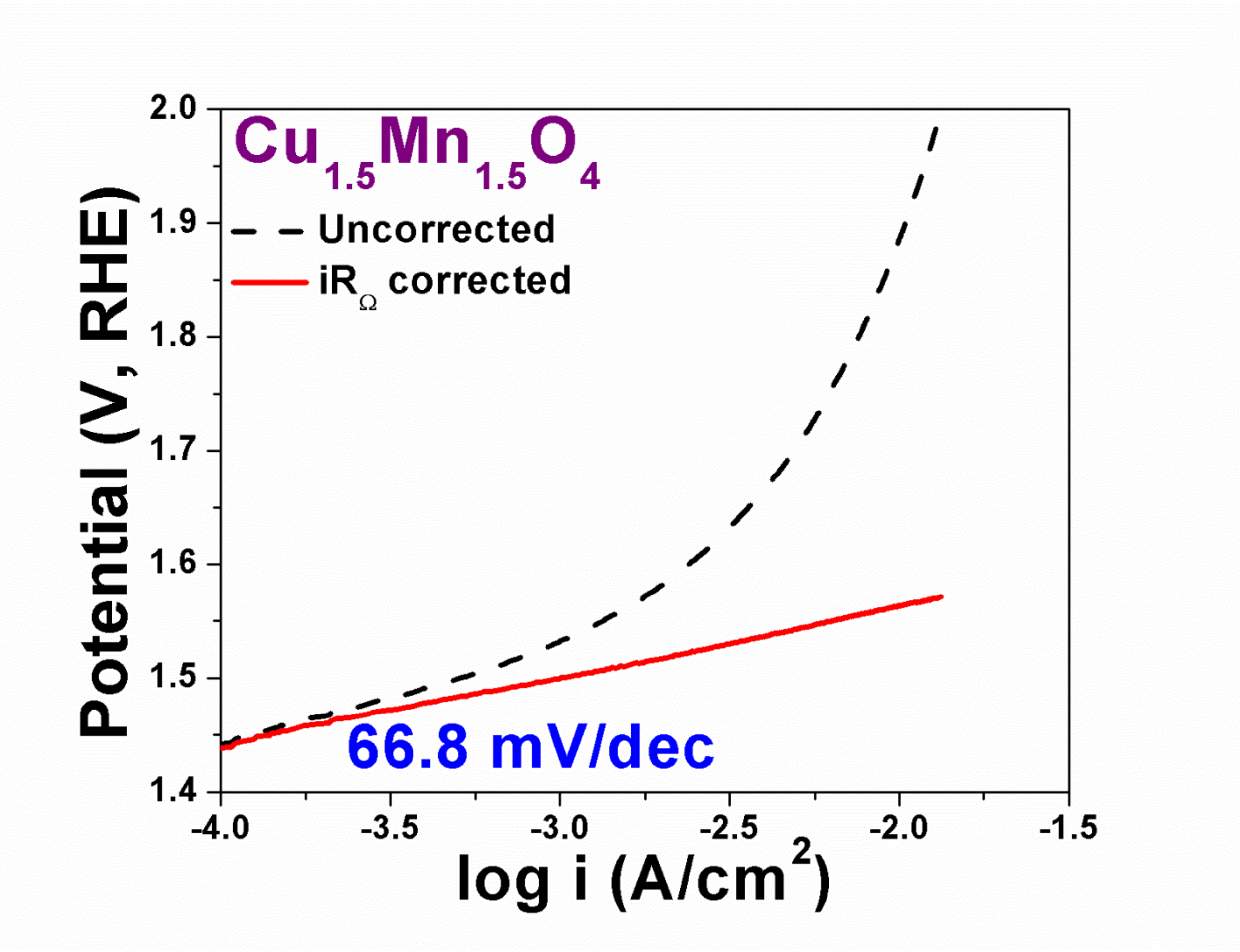

Figure S7. The Tafel plot (for OER) after iR_{Ω} correction of $Cu_{1.5}Mn_{1.5}O_4$

124 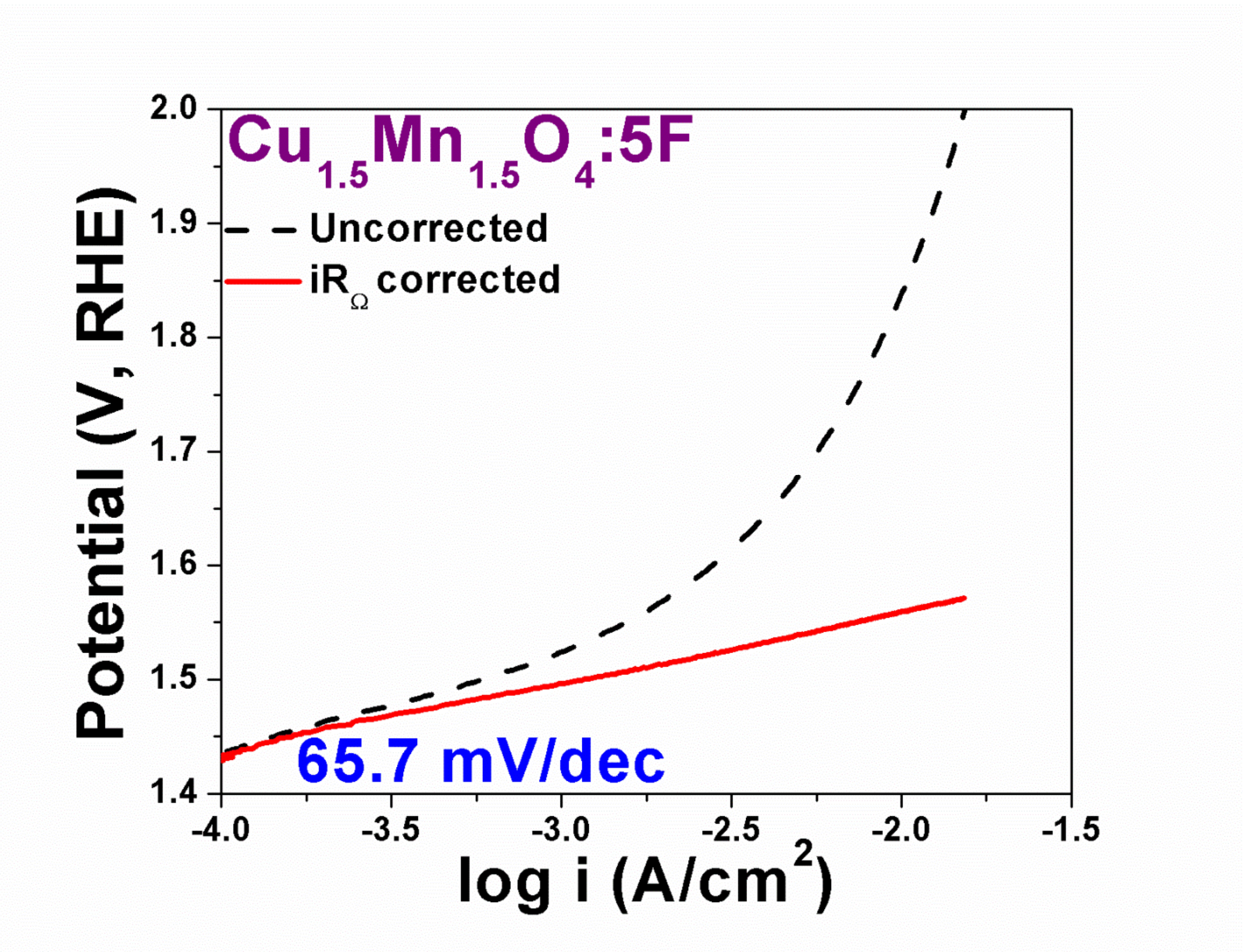

125 **Figure S8.** The Tafel plot (for OER) after iR_Ω correction of $Cu_{1.5}Mn_{1.5}O_4$:5F

126

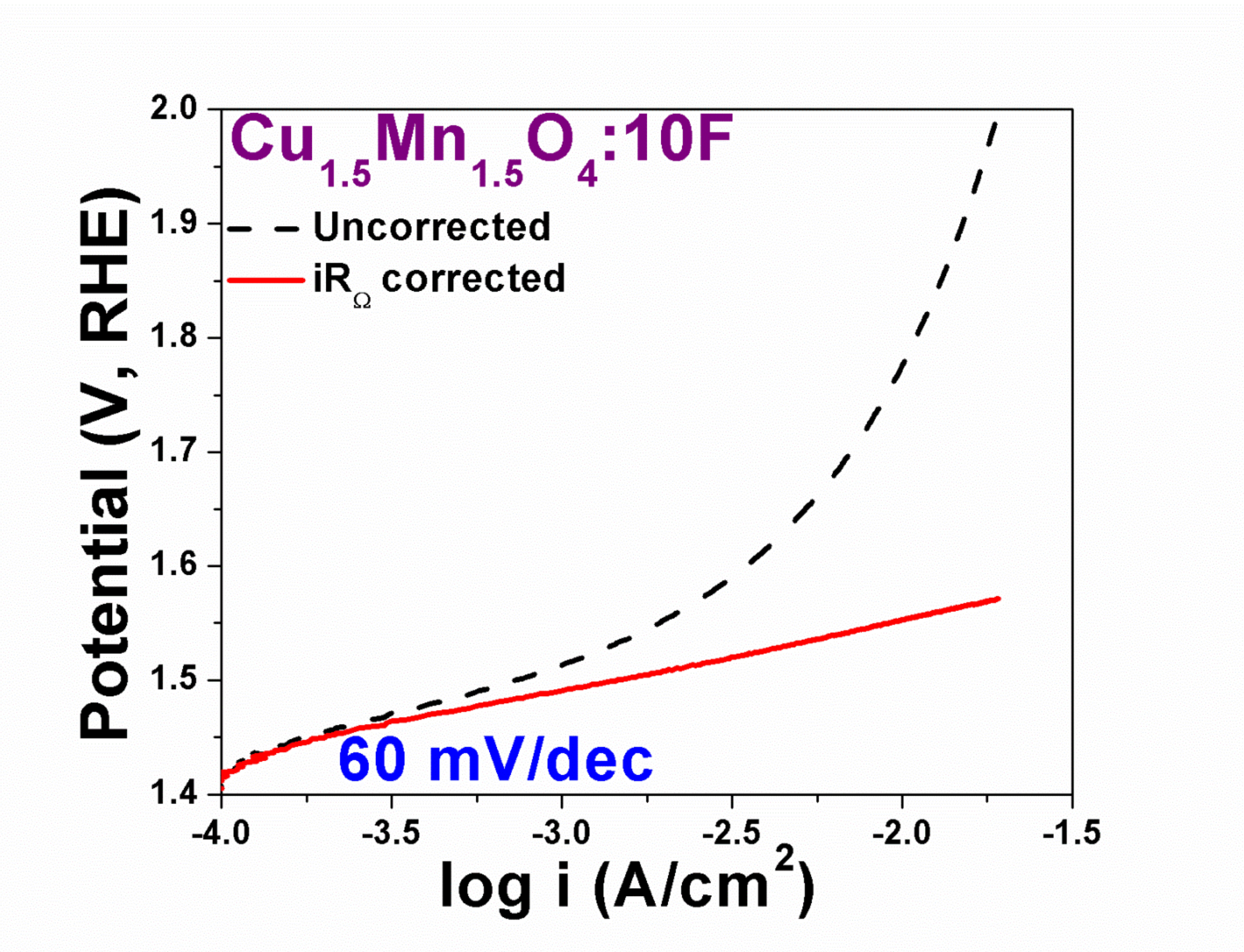

Figure S9. The Tafel plot (for OER) after iR_{Ω} correction of $Cu_{1.5}Mn_{1.5}O_4$:10F

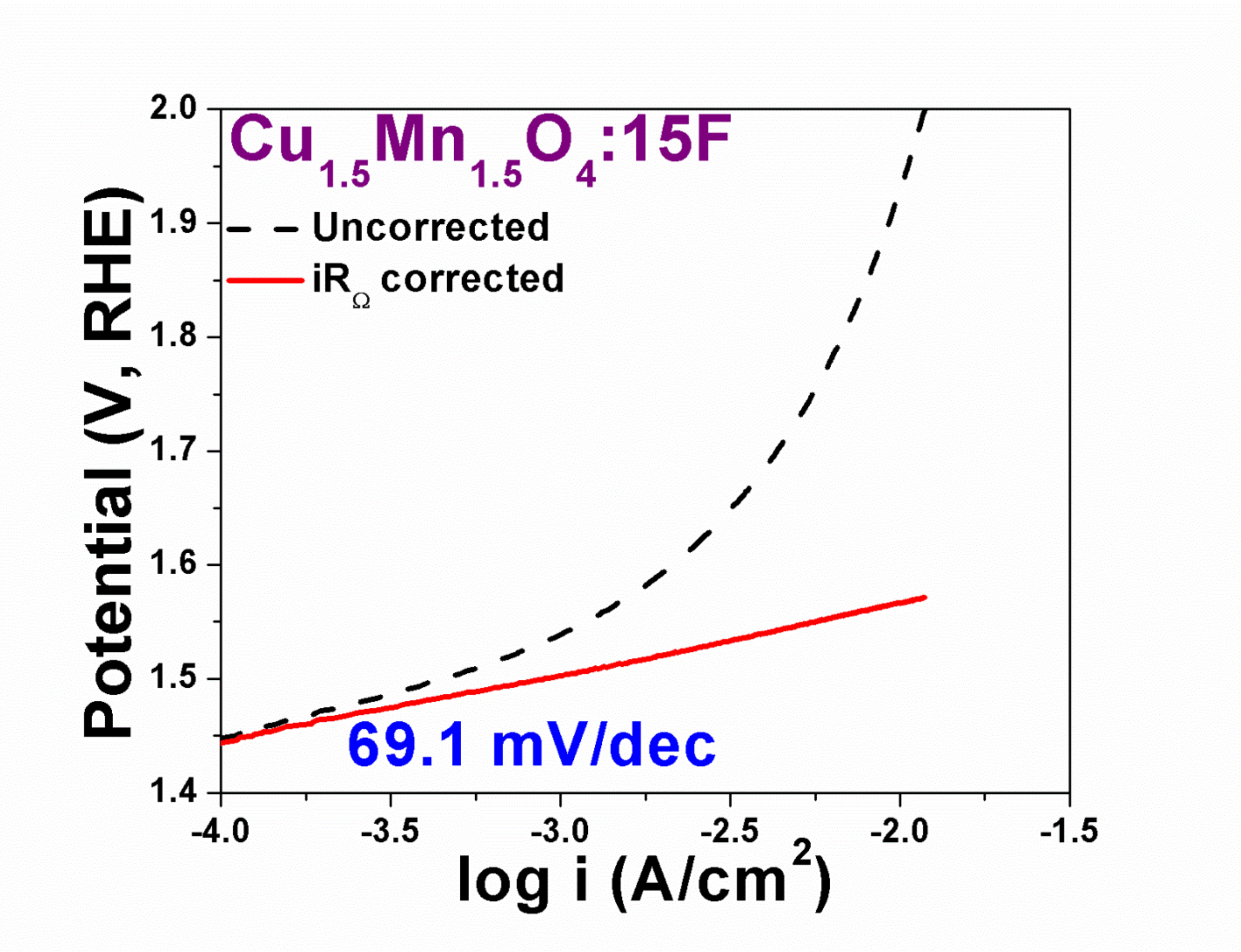

Figure S10. The Tafel plot (for OER) after iR_{Ω} correction of $Cu_{1.5}Mn_{1.5}O_4$:15F

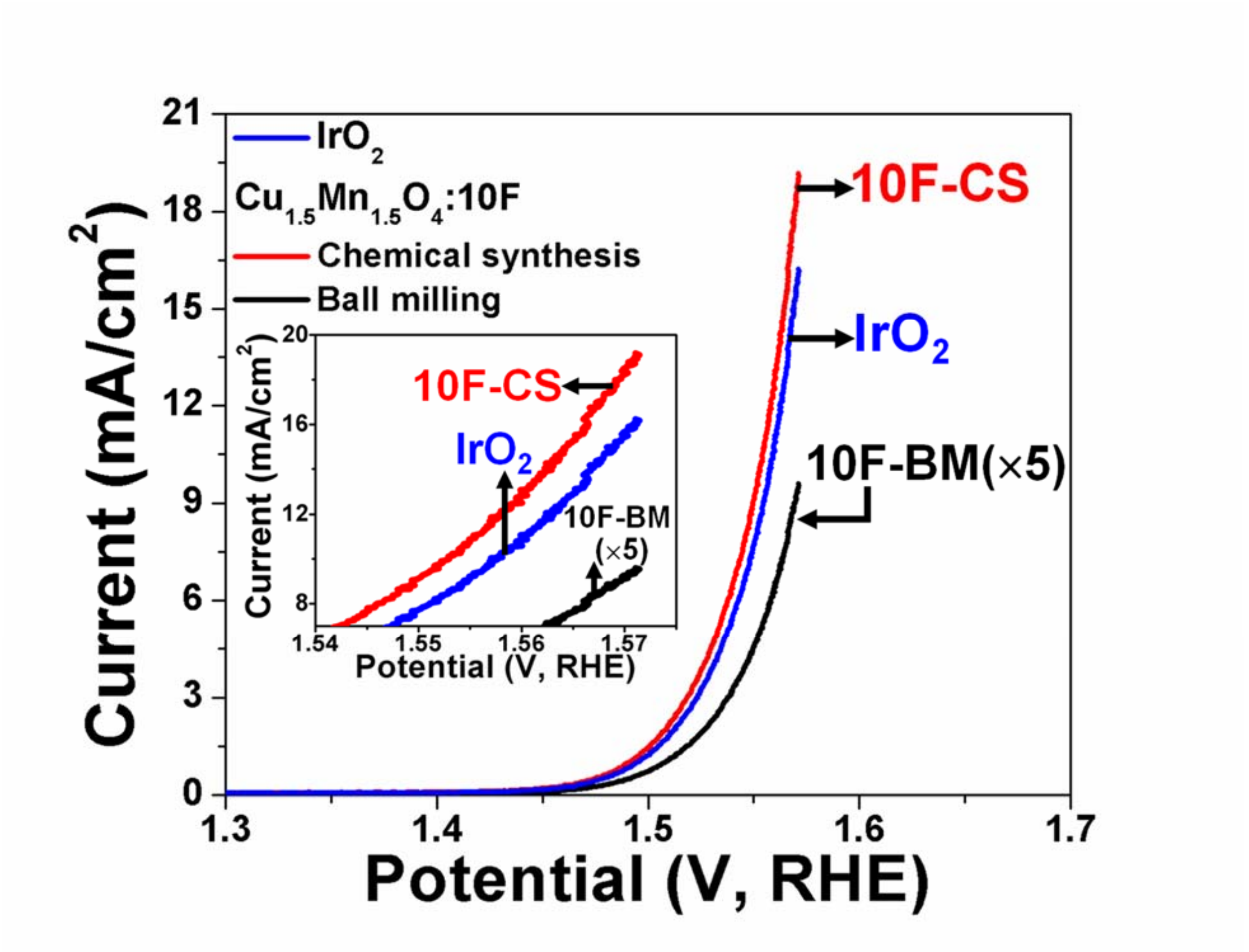

Figure S11. The polarization curve of chemically synthesized and ball-milled $Cu_{1.5}Mn_{1.5}O_4$:10F using total loading of 1 mg/cm^2 and in-house synthesized IrO_2 using total loading of 0.15 mg/cm^2 obtained in 0.5 M H_2SO_4 solution at 40^0C with a scan rate of 5 mV/sec after iR_Ω correction

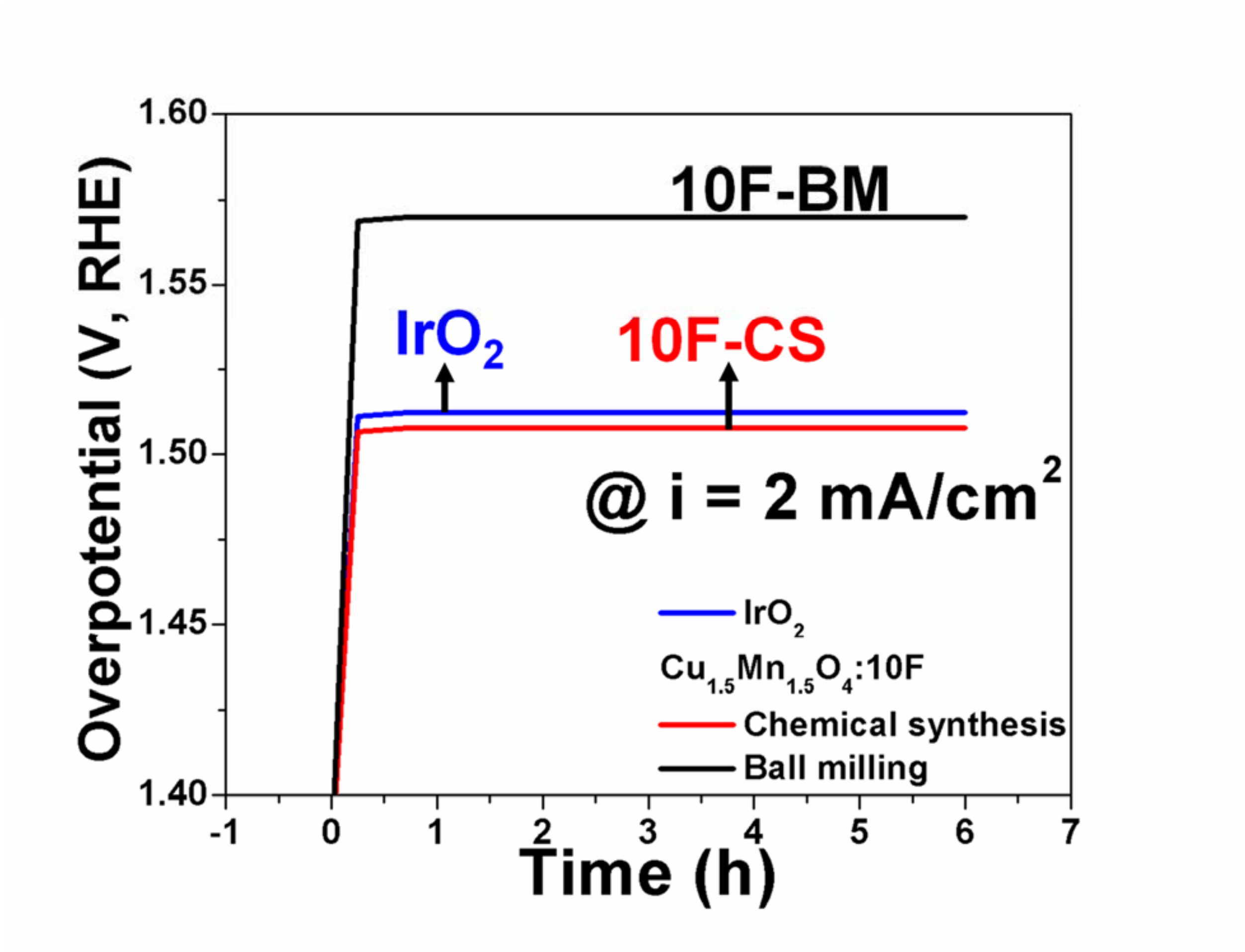

Figure S12. Galvanostatic (constant current) measurement of electrochemical activity of chemically synthesized and ball milled $Cu_{1.5}Mn_{1.5}O_4$:10F (total loading=1 mg/cm^2) and in-house synthesized IrO_2 (total loading=0.15 mg/cm^2) performed in 0.5 M H_2SO_4 electrolyte solution at 40^{0}C at a constant current of ~2 mA/cm^2

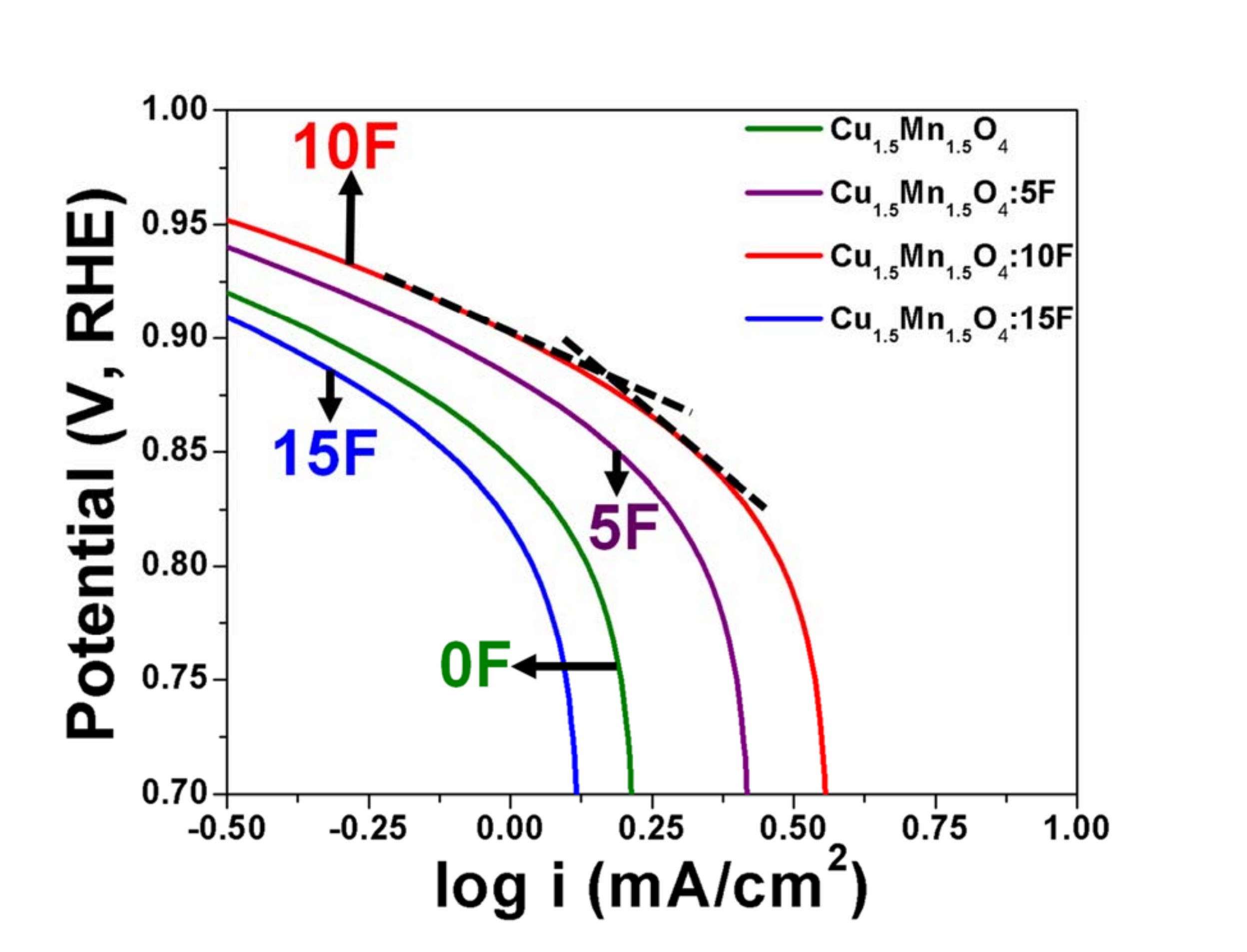

Figure S13. The Tafel plot (for ORR) after iR_{Ω} correction of $Cu_{1.5}Mn_{1.5}O_4$, $Cu_{1.5}Mn_{1.5}O_4$:5F, $Cu_{1.5}Mn_{1.5}O_4$:10F and $Cu_{1.5}Mn_{1.5}O_4$:15F

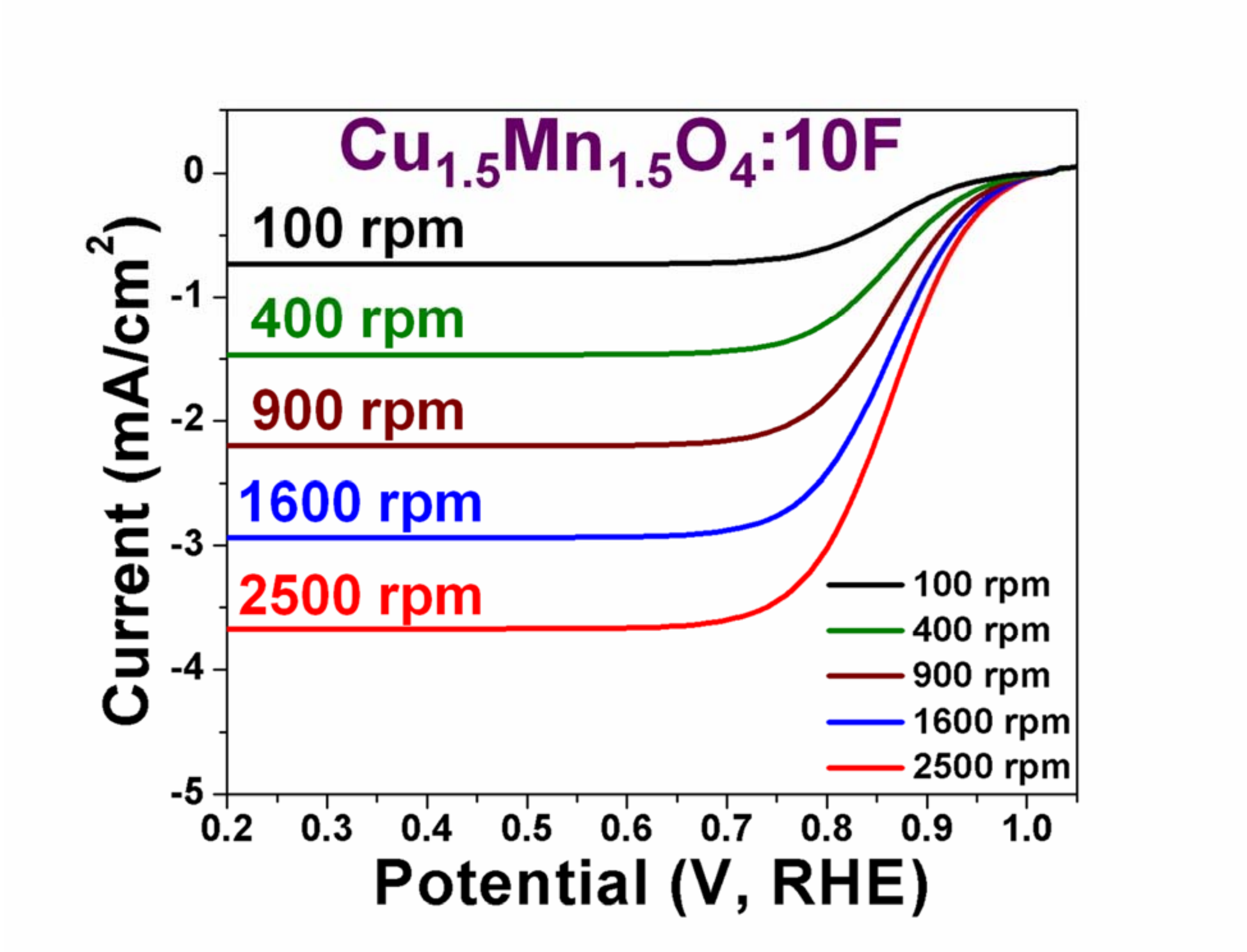

Figure S14. The polarization curve for ORR of $Cu_{1.5}Mn_{1.5}O_4$:10F (total loading = 50 μg/cm^2) at different rotation speeds measured in O_2-saturated 0.5 M H_2SO_4 solution at 26^{0}C with a scan rate of 5 mV/sec

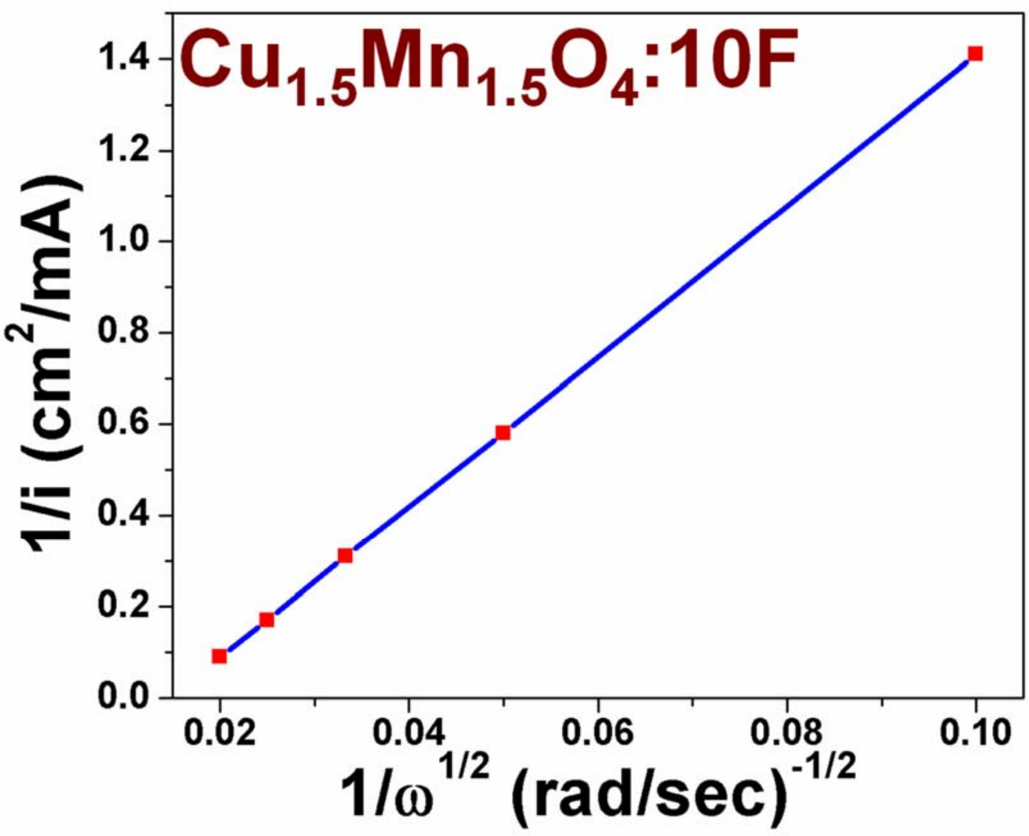

Figure S15. The Koutechy-Levich plot for ORR of $Cu_{1.5}Mn_{1.5}O_4$:10F at ~0.6 V (*vs* RHE)

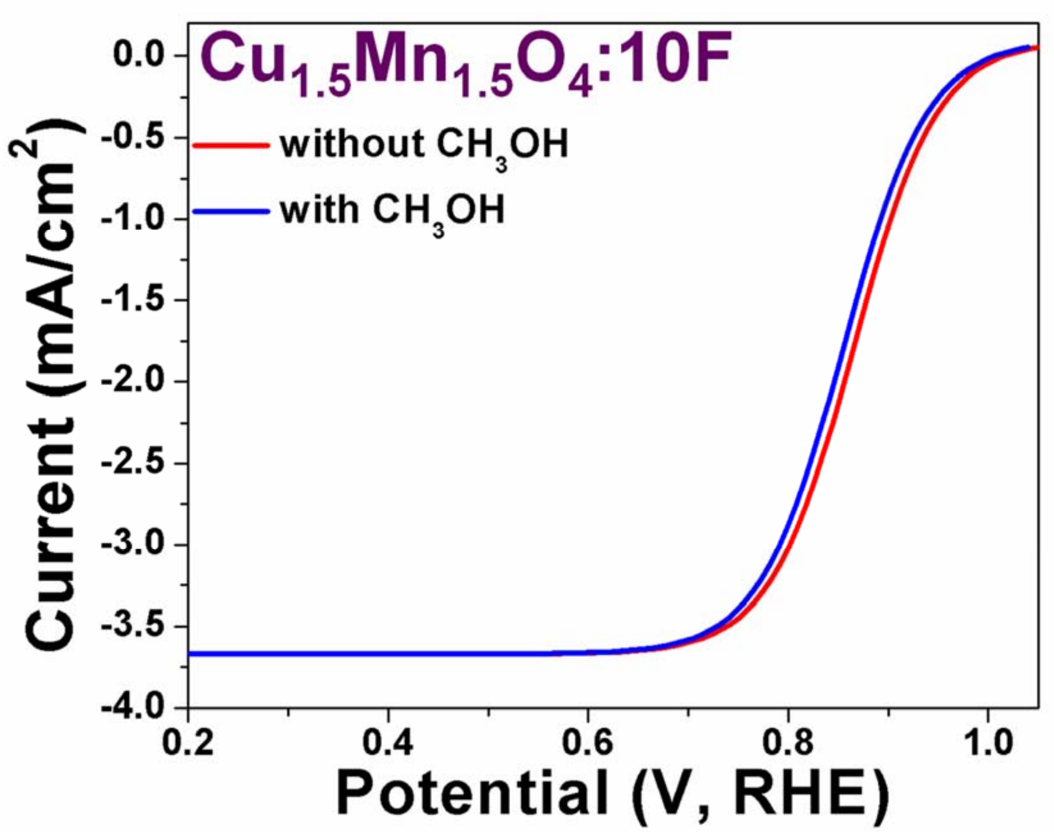

Figure S16. The polarization curve of $Cu_{1.5}Mn_{1.5}O_4$:10F obtained in O_2-saturated 0.5 M H_2SO_4 solution at 26^0C with rotation speed of 2500 rpm and scan rate of 5 mV/sec after iR_{Ω} correction using total loading of 50 μg/cm^2, with and without 1 M methanol in 0.5 M H_2SO_4 electrolyte solution

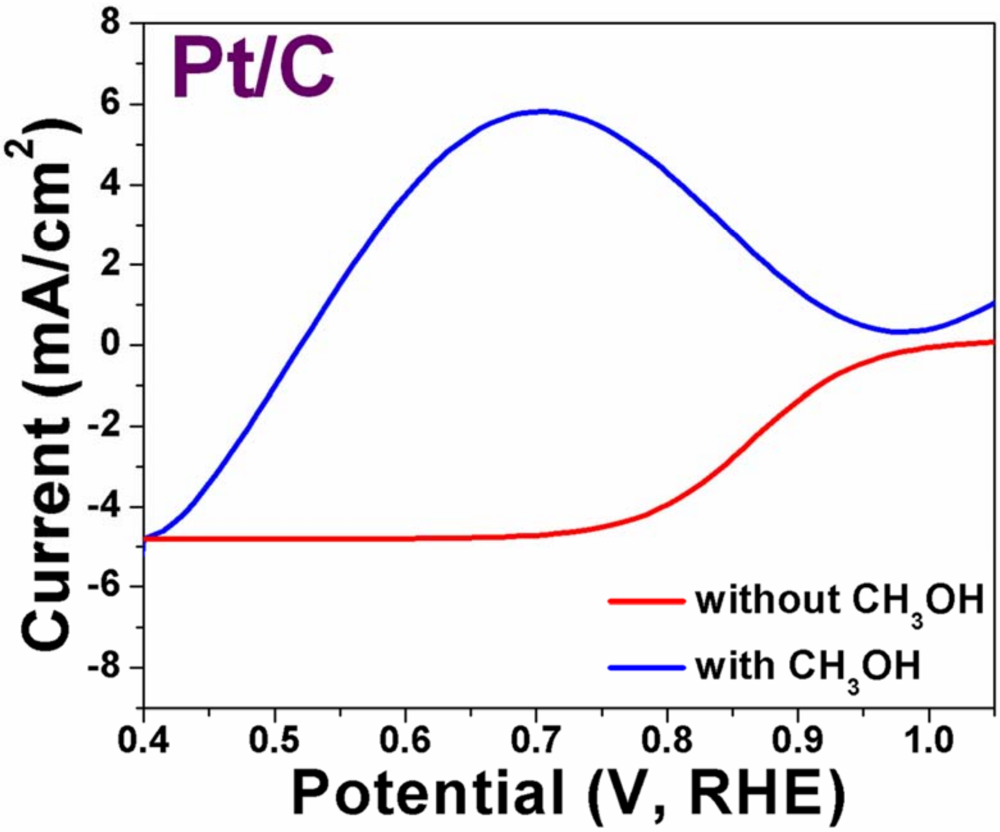

Figure S17. The polarization curves of Pt/C obtained in O_2-saturated 0.5 M H_2SO_4 solution at 26^0C with rotation speed of 2500 rpm and scan rate of 5 mV/sec after iR_{Ω} correction using Pt loading of 30 $\mu g_{Pt}/cm^2$ for Pt/C, with and without 1 M methanol in 0.5 M H_2SO_4 electrolyte solution

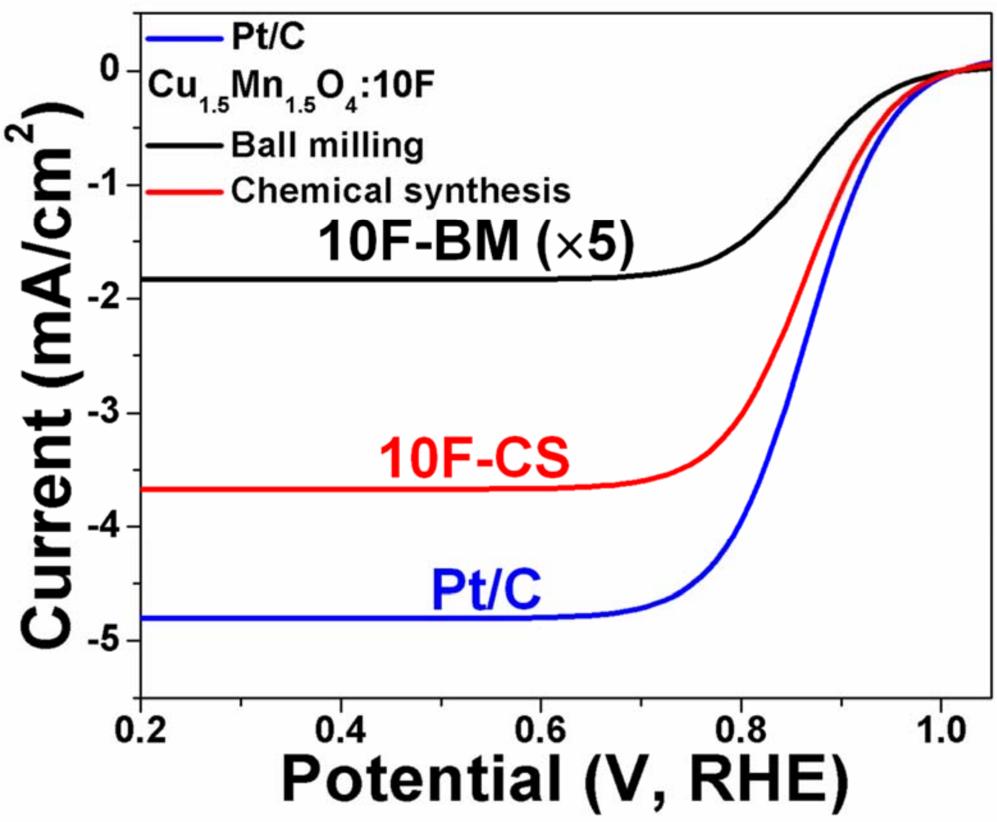

Figure S18. The polarization curves of chemically synthesized and ball-milled $Cu_{1.5}Mn_{1.5}O_4$:10F and Pt/C obtained in O_2-saturated 0.5 M H_2SO_4 solution at 26^0C with rotation speed of 2500 rpm and scan rate of 5 mV/sec after iR_Ω correction using total loading of 50 μg/cm^2 for $Cu_{1.5}Mn_{1.5}O_4$:10F and Pt loading of 30 μg_{Pt}/cm^2 for Pt/C

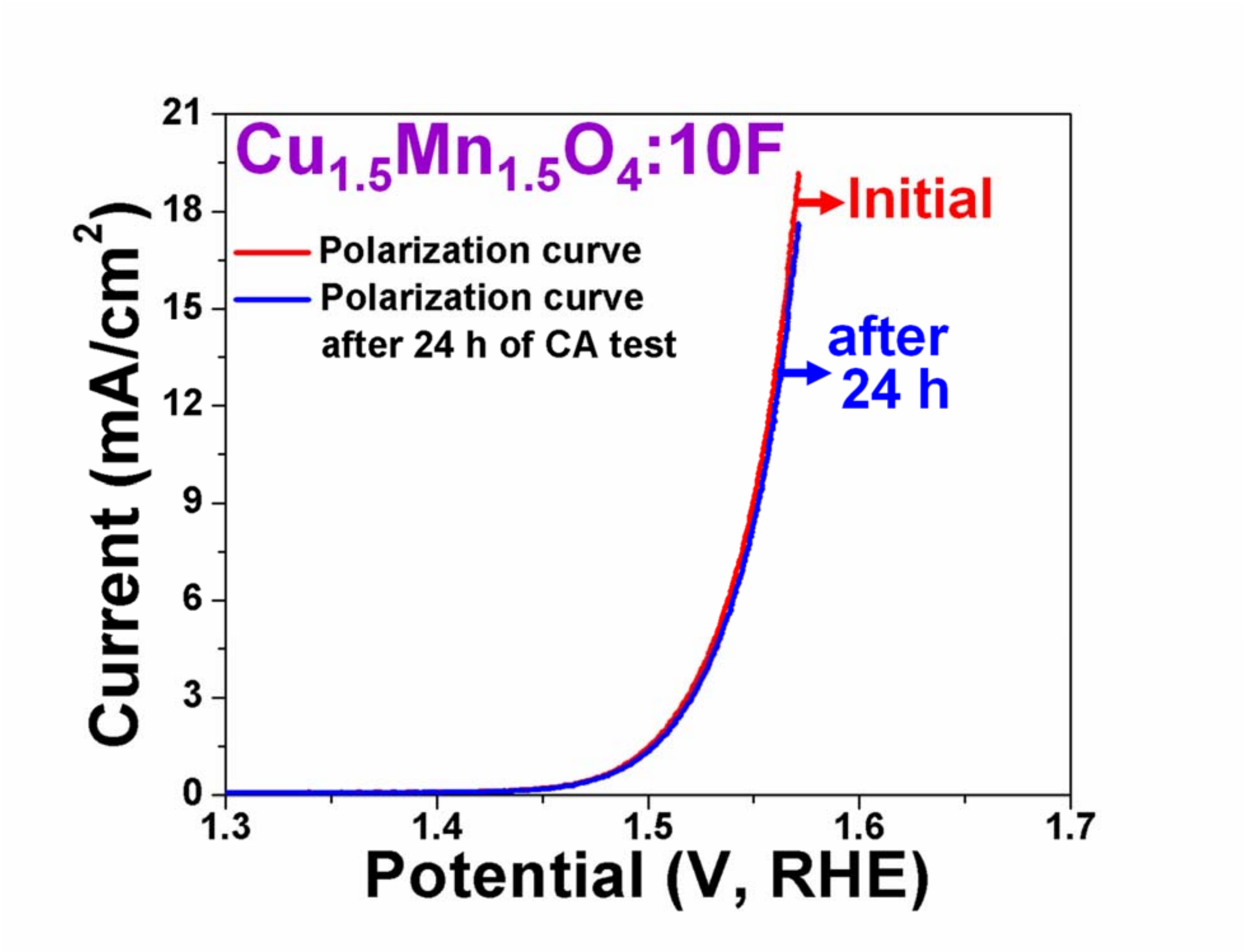

Figure S19. The iR_Ω corrected polarization curve of $Cu_{1.5}Mn_{1.5}O_4$:10F (total loading = 1 mg/cm^2) obtained after 24 h of chronoamperometry test in 0.5 M H_2SO_4 solution at 40^{0}C with a scan rate of 5 mV/sec

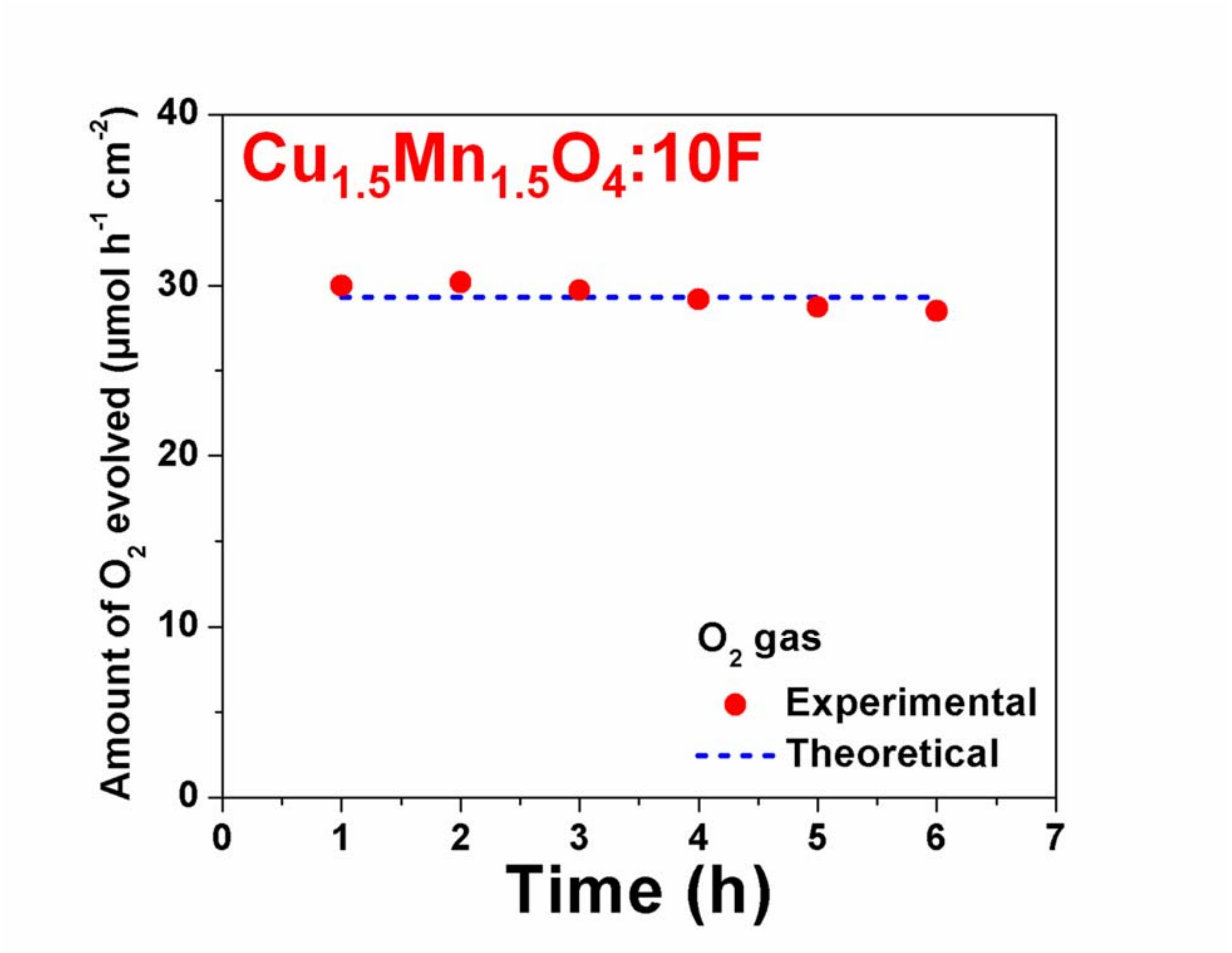

Figure S20. Theoretical and experimentally measured concentration of O_2 gas, measured (for 6 h) during chronoamperometry test of $Cu_{1.5}Mn_{1.5}O_4$:10F (total loading=1 mg/cm^2), performed in 0.5 M H_2SO_4 solution under a constant potential of ~1.55 V (*vs* RHE) at 40^0C

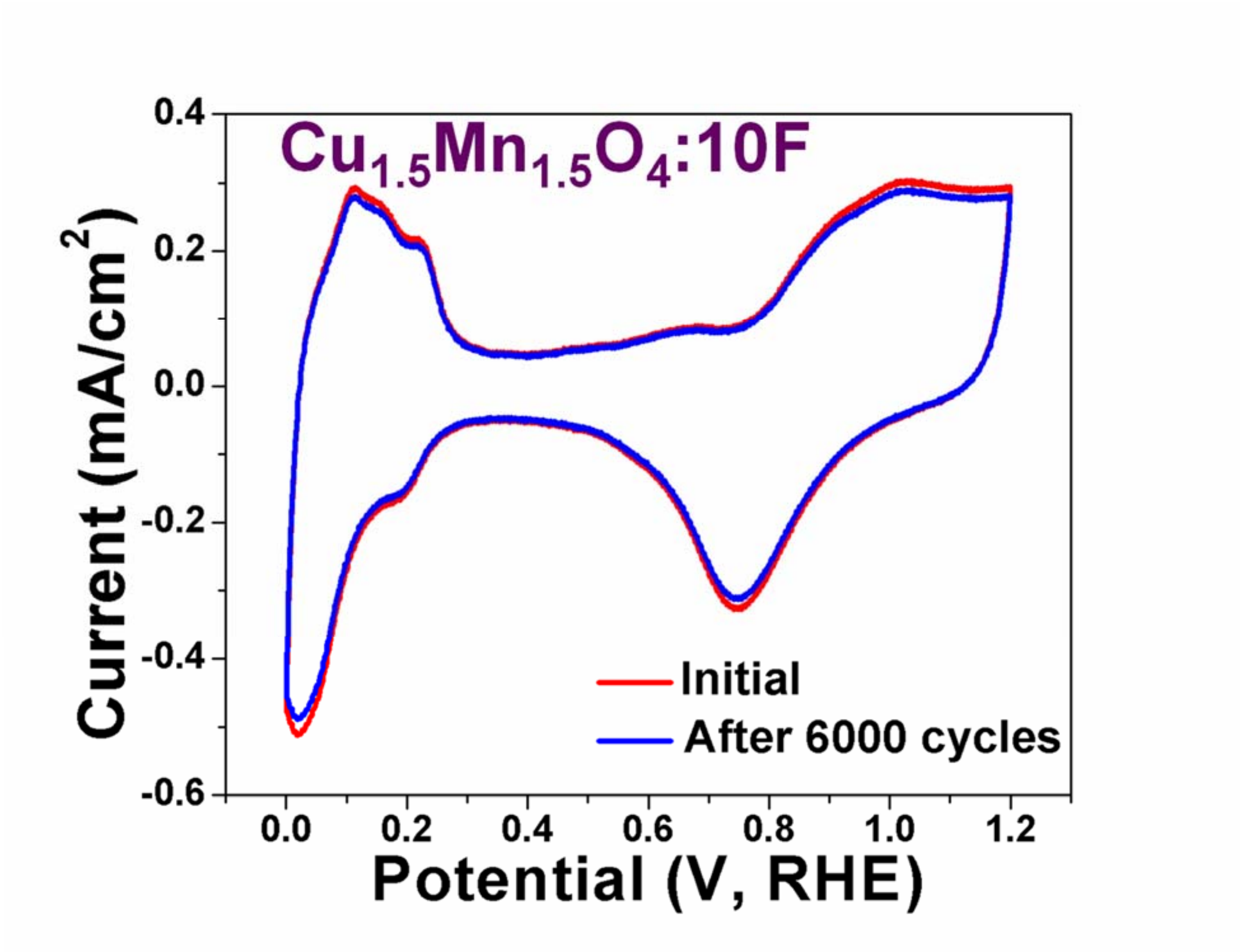

Figure S21. The cyclic voltammogram (CV) of $Cu_{1.5}Mn_{1.5}O_4$:10F measured in N_2 saturated 0.5 M H_2SO_4 at 26^0C at scan rate of 5 mV/sec using total loading of 50 μg/cm^2, initial and after 6000 cycles

References:

1. Lee K, Zhang L, Zhang J. Ir x Co 1− x (x= 0.3–1.0) alloy electrocatalysts, catalytic activities, and methanol tolerance in oxygen reduction reaction. *Journal of Power Sources* **170**, 291-296 (2007).

2. Wang D, *et al.* Facile Synthesis of Carbon-Supported Pd–Co Core–Shell Nanoparticles as Oxygen Reduction Electrocatalysts and Their Enhanced Activity and Stability with Monolayer Pt Decoration. *Chemistry of Materials* **24**, 2274-2281 (2012).
